# Challenging ecogeographical rules: Phenotypic variation in the Mountain Treeshrew (*Tupaia montana*) along tropical elevational gradients

**DOI:** 10.1371/journal.pone.0268213

**Published:** 2022-06-17

**Authors:** Arlo Hinckley, Ines Sanchez-Donoso, Mar Comas, Miguel Camacho-Sanchez, Melissa T. R. Hawkins, Noor Haliza Hasan, Jennifer A. Leonard

**Affiliations:** 1 Conservation & Evolutionary Genetics Group, Estación Biológica de Doñana-CSIC, Seville, Spain; 2 Division of Mammals, Department of Vertebrate Zoology, National Museum of Natural History, Smithsonian Institution, Washington, DC, United States of America; 3 Departamento de Zoología, Universidad de Sevilla, Seville, Spain; 4 Departamento de Zoología, Universidad de Granada, Granada, Spain; 5 Department of Biological Sciences, Dartmouth College, Hanover, NH, United States of America; 6 Instituto Andaluz de Investigación y Formación Agraria, Pesquera, Alimentaria y de la Producción Ecológica (IFAPA), Alcalá del Río, Seville, Spain; 7 Institute for Tropical Biology and Conservation, Universiti Malaysia Sabah, Kota Kinabalu, Sabah, Malaysia; Laboratoire de Biologie du Développement de Villefranche-sur-Mer, FRANCE

## Abstract

Bergmann’s and Allen’s rules were defined to describe macroecological patterns across latitudinal gradients. Bergmann observed a positive association between body size and latitude for endothermic species while Allen described shorter appendages as latitude increases. Almost two centuries later, there is still ongoing discussion about these patterns. Temperature, the common variable in these two rules, varies predictably across both latitude and elevation. Although these rules have been assessed extensively in mammals across latitude, particularly in regions with strong seasonality, studies on tropical montane mammals are scarce. We here test for these patterns and assess the variation of several other locomotory, diet-associated, body condition, and thermoregulatory traits across elevation in the Mountain Treeshrew (*Tupaia montana*) on tropical mountains in Borneo. Based on morphological measurements from both the field and scientific collections, we found a complex pattern: Bergmann’s rule was not supported in our tropical mountain system, since skull length, body size, and weight decreased from the lowest elevations (<1000 m) to middle elevations (2000–2500 m), and then increased from middle elevations to highest elevations. Allen’s rule was supported for relative tail length, which decreased with elevation, but not for ear and hindfoot length, with the former remaining constant and the latter increasing with elevation. This evidence together with changes in presumed diet-related traits (rostrum length, zygomatic breadth and upper tooth row length) along elevation suggest that selective pressures other than temperature, are playing a more important role shaping the morphological variation across the distribution of the Mountain Treeshrew. Diet, food acquisition, predation pressure, and/or intra- and inter-specific competition, are some of the potential factors driving the phenotypic variation of this study system. The lack of variation in body condition might suggest local adaptation of this species across its elevational range, perhaps due to generalist foraging strategies. Finally, a highly significant temporal effect was detected in several traits but not in others, representing the first phenotypic variation temporal trends described on treeshrews.

## Introduction

Bergmann’s [1] and Allen’s [2] ecogeographical rules describe geographical variation of morphological traits primarily in endotherms. Bergmann’s Rule describes a pattern of individuals being larger in colder habitats. Bergmann [1] explained this trend through the heat conservation hypothesis: in colder climates (higher latitudes) larger animals, with a lower surface-area-to-volume ratio, increase their ability to conserve heat. Allen’s Rule [2] describes a pattern of individuals having shorter appendages in colder habitats (higher latitudes). The reduction of surface area to volume ratio through a decrease in appendage length increases thermal efficiency.

Although these rules were initially developed based on patterns across latitudinal gradients, they were later extensively tested across elevational gradients (e.g., [3–7]). Elevational gradients share certain properties with latitudinal gradients, including the key change in temperature [4]. Common characteristics of elevational gradients (e.g. decrease in temperature and oxygen levels as altitude increases) are consistent across large geographic scales, enabling the study of the influence of environmental variables in natural populations across numerous replicated systems. In contrast to latitudinal gradients, elevational gradients exhibit drastic environmental transitions at spatial scales that are small relative to the dispersal capability of many species, making these optimal to study whether adaptive divergence is possible when confronted with gene flow [4].

Many studies have tested these rules in mammals both within and between species, and in different habitats (e.g. [5, 8–13]). Some mammals have patterns consistent with these predictions (e.g. [3, 14–18]) while others do not (e.g. [3, 7, 15, 17, 19–24]). However, there are multiple biases in the literature. There is a tendency to test these rules in species that are known to have a high/moderate degree of morphological differentiation across their geographical distribution and ignore those that do not [10]. There is also a strong bias towards studies in habitats with a strong temperature seasonality, especially the Holarctic (e.g. [5, 7–9, 18, 24–26]). This bias is unfortunate because seasonality by itself likely affects the evolution of body size [27–30].

Despite the valuable insights obtained from elevational approaches, few studies have tested these rules in mammals along elevational gradients in tropical areas with little seasonality [31, 32]. Indeed, the few studies from tropical regions seem to refute Bergmann’s rule and provide mixed support for Allen’s rule. A negative effect of latitude on body size was found both in the Common Treeshrew in tropical Asia (*Tupaia glis*; [23]), and in peccaries in the American tropics (Tayassuidae; [33]) indicating the inverse of Bergmann’s rule. Bergmann’s rule was not supported either at the interspecific nor at the intraspecific level along elevational gradients in New Guinea for rats (*Rattus* sp.; [31]) or forest passerines [34]. At an interspecific level, Bergmann’s rule was not supported in Bornean birds, which decrease in mass with elevation, while Allen’s rule had mixed support, with high-elevation communities exhibiting narrower, shorter and relatively smaller bills but relatively longer tarsi [35]. Neotropical Torrent ducks (*Merganetta armata*) followed Bergmann’s rule across latitude, but not along elevation, while Allen’s rule was neither supported across latitude nor elevation, showing an opposite trend for wing length [36].

Vegetation zonation can be expected to have an important impact on habitat structure and resource availability, both of which might pose strong selection pressures on local populations [37]. Populations might adapt to these different habitats through changes in locomotion, diet-associated traits, and/or insulation (changes in fat levels and/or fur density/length to alter heat loss; [6, 31, 35]). If populations are well adapted to these habitats, limited variation in body condition should be expected along elevational gradients [4]. Nevertheless, local adaptation must be constrained to a certain degree since species usually have restricted elevational distributions [4].

A likely explanation for the few intraspecific studies of morphological variation across elevational gradients in tropical habitats without a strong temperature seasonality is the lack of species with an appropriate distribution to test this, since species in the tropics tend to have narrow elevational ranges [38]. The Mountain Treeshrew (*Tupaia montana*) provides a rare opportunity to test Bergmann’s and Allen’s rules along wide tropical elevational gradients. These are one of the few Bornean mammals distributed along a wide elevational gradient (800–3400 m) on some of the tallest tropical mountains in tropical east Asia, including Mt. Kinabalu [39].

Here we assess phenotypic variation across an elevational gradient (in which elevation is treated as a surrogate of temperature) in the tropics in the Mountain Treeshrew, an endemic Bornean species. We combine field and museum data to test Bergmann’s rule (increasing size with elevation using weight, head-and-body length, and skull length); Allen’s rule (relatively smaller appendages with increasing elevation using ear, hindfoot, and tail length as a proportion of head-and-body length); changes in body condition as a surrogate of lipid content which is frequently assumed to be directly positively linked to fitness; trophic variation using rostrum length, upper toothrow length, and zygomatic breadth as a proportion of skull length; changes in locomotor behavior using hindfoot, and tail length as a proportion of head-and-body length; and insulation using guard hair length.

## Materials and methods

### Model species

The Mountain Treeshrew (*Tupaia montana*) is a terrestrial/ scansorial member of the Order Scandentia that has an omnivorous diet [40, 41]. It is an endemic Bornean species and montane specialist, distributed above 800 m in the highlands of northern Borneo. It has been recorded in all four vegetation zones described on Mt. Kinabalu [42, 43], but it is rare in the lowland forest (< 1,200 m) and becomes much more common through the lower montane (1,200–2,000 m) and upper montane forest (around 2,000–2,800 m) where it reaches highest densities, and in the subalpine forest (2,800–3,400 m) where its population density possibly decreases again [39].

### Data collection

**Fig 1:**
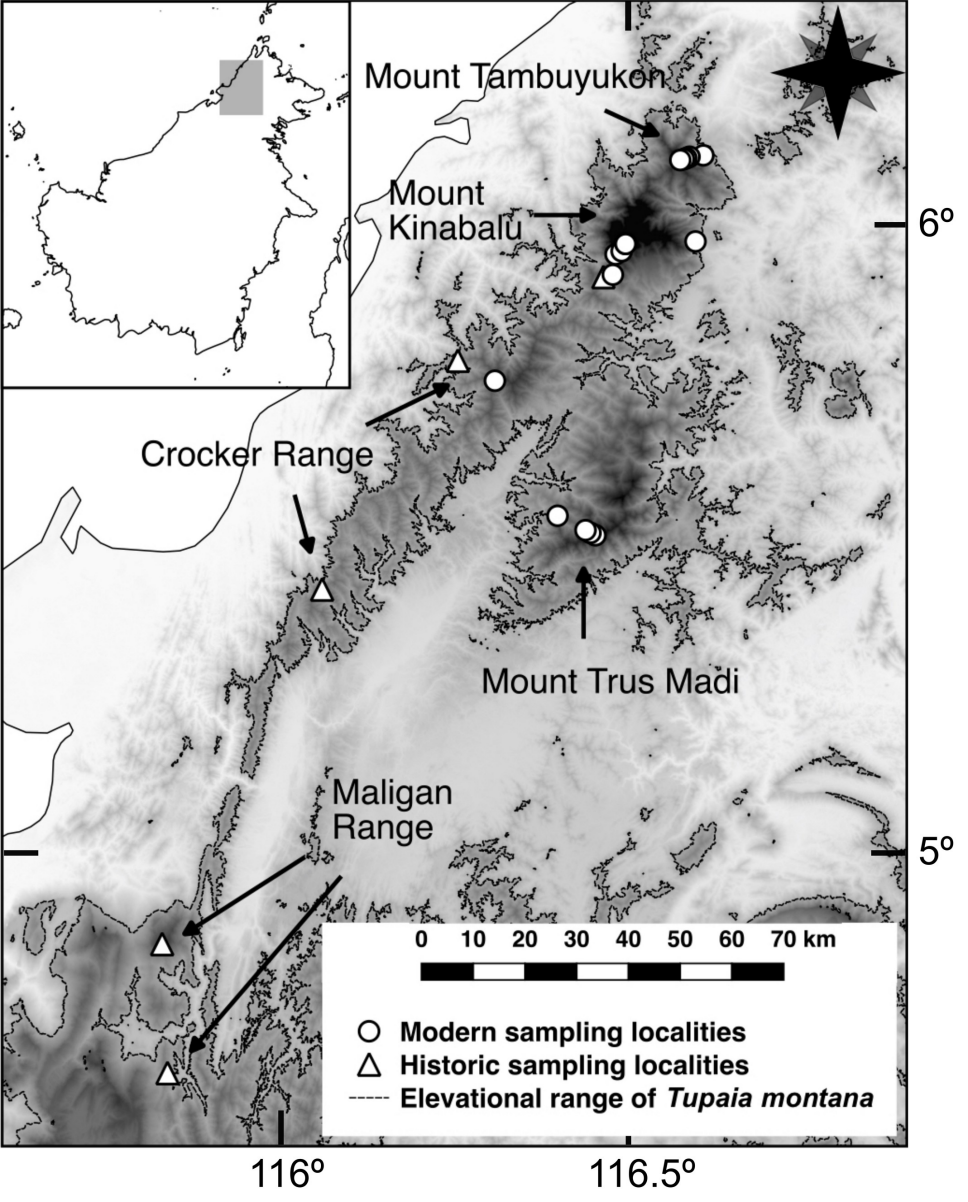
Physical map of study area. Map of northern Borneo showing mountains and potential distribution of Mountain Treeshrews based on its elevation range (areas above 800 m). Areas between 800 and 1600 m are highlighted in light gray, those between 1600 and 2400 m in dark gray and those over 2400 m in black.

Mountain Treeshrews were sampled in the field along elevational gradients on Mt. Kinabalu (4,095 m; 6.07° N 116.56° E), Mt. Tambuyukon (2,579 m; 6.20° N 116.66° E), Mt. Alab, Crocker Range (2,050 m; 5.83° N, 116.34° E), and Mt. Trus Madi (2,643 m; 5.55° N, 116.52° E) (**Fig 1**, [39]). We used Tomahawk and Sherman live-traps baited with banana, dry fish, shrimp paste with rice flour, coconut, and/ or palm nuts. Trapping was performed following ethical standards according to the guidelines of the American Society of Mammalogists [44]. Animal care and use committees approved the protocols (Smithsonian Institution, National Museum of Natural History, proposal number 2012–04 and Estación Biológica de Doñana proposal number CGL2010-21424). Field research was approved by Sabah Parks (Refs: TS/PTD/5/4 Jld. 45 (33), TS/PTD/5/4 Jld. 47 (25), TK/PP:5/78 Jld 5), the Economic Planning Unit (Ref: 100-24/1/299), Sabah Wildlife Department (Ref: JHL: (HQ):100-42/ 1 JLD.27), Sabah Forestry Department [Refs: JPHTN/TP(FSP) 100-14/18/2/KLT.31(31) & JPHTN/TP(FSP) 100-14/18/2/KLT.31(17)] and the Sabah Biodiversity Council/Centre (Ref:TK/PP:8/8Jld.2). Two hundred thirty-three Mountain Treeshrews were captured from 836 to 3,382 m during three field seasons (2012, 2013 and 2016). We sampled a total of 81 unique individuals (ear clipping allowed identification of recaptured individuals) in 1737 trap-nights on Mt. Kinabalu (0.047 individuals per trap-night), 103 in 4369 trap-nights on Mt. Tambuyukon (0.023 individuals per trap-night), 9 in 960 trap-nights on Mt. Alab (0.009 individuals per trap-night), and 40 in 1600 trap-nights on Mt. Trus Madi (0.025 individuals per trap-night) (**S1 Table**, individual BOR numbers as in [39]). These individuals constituted the Estación Biológica de Doñana collection (BOR and EBD-CSIC numbers, for released and collected specimens). We weighed animals with Pesola® 300 g with ± 0.3% precision scale. We measured body length as the distance from the tip of the snout to the base of the tail, with the ruler held along the dorsum, to the nearest 1 mm (head-and-body length, HBL); tail length (TL), from posterior margin of the anus to tail tip, excluding hairs; hindfoot length (HFL), from back of the heel to tip of the longest digit, including the claw; and ear length (EL), from notch at the base of the ear to distal edge. These measures were taken immediately after specimens were collected (or from live specimens in **S1 File** analyses). Sex was determined from external sexual organs, and in the case of collected animals, also through dissection and discrimination between ovaries, testes and immature gonads during specimen preparation. After specimen preparation, the following craniodental measurements were collected on the cleaned and dried skulls with a Fowler High Precision electronic digital caliper to the nearest 0.01 mm by A. H. and as described in [23]: condyloincisive length (CIL), as the greatest distance between anterior‐most surface of I1 and caudal surface of occipital condyle; zygomatic breadth (ZB), as the greatest distance between lateral surfaces of zygomatic arch; upper toothrow length (UTL), as the greatest distance between anterior‐most surface of I1 and posterior‐most surface of M3; and rostrum length (RL), as the greatest distance between anterior most surface of premaxilla and anterior most surface of lacrimal foramen (**Fig 1 in S1 File**). We measured the right side for all bilateral measurements.

To complement our field data, specimen-label-associated measurements were incorporated from the collections of the Field Museum of Natural History (N = 33, from 1937 and 1965), Harvard Museum of Comparative Zoology (N = 72, collected in 1937), and Sabah Museum (N = 88, from 1971 to 2007), while skull and specimen-label-associated measurements were taken from United States National Museum of Natural History (N = 72, collected in 1951, 1953 and 1961) and Natural History Museum of London (N = 25, collected in 1952 and 1961). An additional mountain, Maligan Range (1953 m; 4.84° N, 115.76° E) was added to this study based only on museum data (**S1 Table**). In total, data from 523 individuals collected between 1937 and 2016 were evaluated (**S1 Table**).

We aged (adult/ non-adult) individuals from museum specimens based on gonad examination in necropsies of wet specimens or on the permanent dentition eruption and lack of deciduous teeth [45]. Individuals that were released in the field were aged based on HBL, body weight and the presence of external sexual organs.

We generated five datasets. The first dataset (D1) included weight measurements of museum specimen adults aged based on gonad examination or on the confirmation of their permanent dentition (N = 84). The second dataset (D2) included HBL and TL measurements of the subset of D1 from the EBD-CSIC collection (N = 51 for HBL, N = 52 for TL). The third dataset (D3) was a subset of D2 that included HFL and EL measurements from adults aged based on their dentition or gonads measured by a single collector (MTRH, N = 35 for HFL, N = 34 for EL). With the creation of D2 and D3 we tried to reduce potential observer bias, live/postmortem stage variation, and ontogeny-associated errors [46, 47]. The fourth dataset (D4) included external measurements (weight, HBL, TL, and EL) of adults aged by any of the methods previously described plus all individuals from Sabah Museum collection, for which we did not have information regarding age (N = 374, pregnant females were excluded). The fifth dataset (D5) included HFL measurements taken including nails (following the U.S.A. procedure: only those coming from EBD-CSIC and U.S.A. collection specimens) from adults aged by any of the methods previously described (N = 167). The analyses of dataset D4 and D5 are included in the **S1 File** as complementary evidence to the main text findings. The sixth dataset (D6) was composed of skull measurements of 136 adult individuals aged based on dentition from several collections and taken by A. H.

Finally, we measured hair length on the dorsum at the rump (HRL) and scapula (HSL) in 17 adult dry skins from Mt. Tambuyukon in order to examine potential pelage differences along elevation. We followed [48] and measured under a magnifier lamp overfur length on the dorsum near the rump and scapula levels by placing a ruler at a right angle to the skin surface and recording the approximate mark where ends of the bunched hairs rested. As [48] pointed out, “the technique is unsophisticated and the results imprecise, but still provide a descriptive estimate of lengths for those pelage constituents”. Pictures of the pelage were taken of several specimens with a Zeiss SteREO Discovery.V8 at 5X to exhibit graphically the conspicuous changes found in hair density and hair thickness along elevation (**S1 File**).

### Inclusivity in global research

Additional information regarding the ethical, cultural, and scientific considerations specific to inclusivity in global research is included in the Supporting Information (**S2 File**)

### Statistical analyses

To test Bergmann’s rule, we tested the effect of elevation over the following variables, as proxies of body size: weight (W), head-and-body length (HBL), and condyle-incisive length (CIL). We fit a linear model for each one of the cited variables. We standardized ‘elevation’ to include it as a quadratic term in the models of W and CIL given that these variables followed a U-shaped distribution along elevation in a preliminary plot. Standardization was done by subtracting the mean of ‘elevation’ and by dividing by its standard deviation (we used the function scale() to standardize ‘elevation’). ‘Elevation’ was added as a linear and non-standardized term in the HBL model after showing HBL followed a linear relationship with elevation. ‘Sex’ was included as a factor of interest in all the models to test for sexual dimorphism. All models initially also included ‘mountain’ and ‘year’ of collection to control for their effect, but these two variables were excluded from the final models when their effect over the response variable was not significant (see final model formulation in **S1 File**). Homoscedasticity and normality of residuals were checked by visual exam of scatterplots. Significance was evaluated with an *F*-test with the function Anova() available in the R car package [49]. Pairwise comparisons between sexes were done with the Tukey post hoc test using the function emmeans() from the emmeans package [50]. We analyzed D4 and D5 in the same manner (**S1 File**). All statistical analyses and graphs were done in R 4.0.3 [51], using RStudio Version 1.3.959 [52].

To test Allen’s rule, we tested the effect of elevation over relative tail length (TL), hindfoot length (HFL), and ear length (EL). We also investigated variation of diet-associated traits across elevation by analyzing zygomatic breadth (ZB), rostrum length (RL), and upper tooth-row length (UTL). We fit Linear Models for each one of the cited variables. ‘Elevation’ was standardized and included as a quadratic term in the models of TL and UTL, and as a linear and non-standardized term in the models of HFL, EL, RL, and ZB as explained above. ‘Sex’ was included in all the models to test for sexual dimorphism, as well as ‘mountain’ and ‘year’ to control for their effect. These two last variables were excluded from the final models when their effect over the response variable was not significant (see final model formulation in **S1 File**). To account for body size and control for allometric covariance, we also included HBL in the models of TL, HFL, and EL, and CIL in the models of RL, ZB, and UTL. Model quality checking, evaluation of significance, pairwise comparisons between sexes, and analyses over D4 and D5 were performed as in Bergmann’s rule analyses (S1 Table; S1 File).

We tested for changes in individual body condition along elevation with the Scaled Mass Index (SMI), following [53, 54]. SMI adjusts the mass of all individuals to the mass they would have if they had the same body size. The SMI was computed for each individual of D2 as follows: SMI = W_i_*(HB_0_/HB_i_)^b^; where W_i_ and HB_i_ are the weight and the head-and-body length of the individual, respectively, HB_0_ is the arithmetic mean value of head-and-body for the whole study population, and *b* is the slope estimate of a standardized major axis (SMA) regression of log-transformed weight on log-transformed head-and-body (*b* = 1.8399). We fit a Linear Model with SMI as response variable, ‘elevation’ and ‘sex’ as explanatory variables of interest, and ‘mountain’ and ‘year’ as factors to control for. Model quality checking and evaluation of significance were performed as previously described.

## Results

### Bergmann’s rule

**Fig 2:**
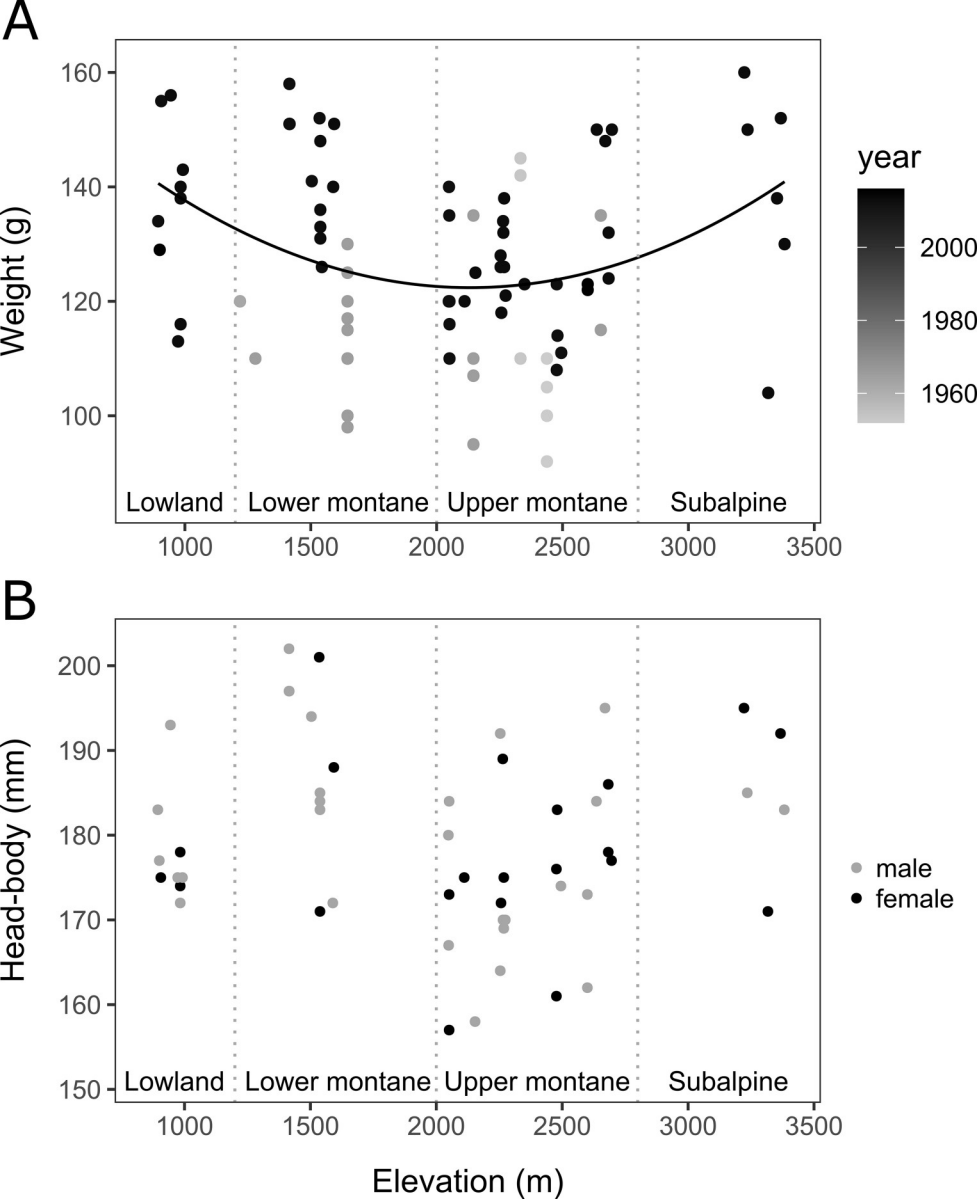
Elevational distribution of body size. Distribution of body size, approximated by (A) weight (W, in g) for D1 individuals and (B) head-and-body length (HBL, in mm) for D2 individuals across the studied elevational gradient. Vegetation zonation is indicated with vertical dotted lines. (A) Dot relative shading corresponds to the year of collection. Weight (W) followed a significant U-shaped distribution along elevation (*P* = 0.0337), with no significant differences between sexes (*P* = 0.8292), but a significant trend to increase over the years (*P*<0.0001) (W model *R*^*2*^_*adj*_ = 0.2498, *F*_4,79_ = 7.909). (B) Black dots represent females, gray dots represent males. Head-and-body length (HBL) was not significantly affected by any of the response variables studied (HBL model *R*^*2*^_*adj*_ = -0.0326, *F*_4,79_ = 7.909).

**Fig 3:**
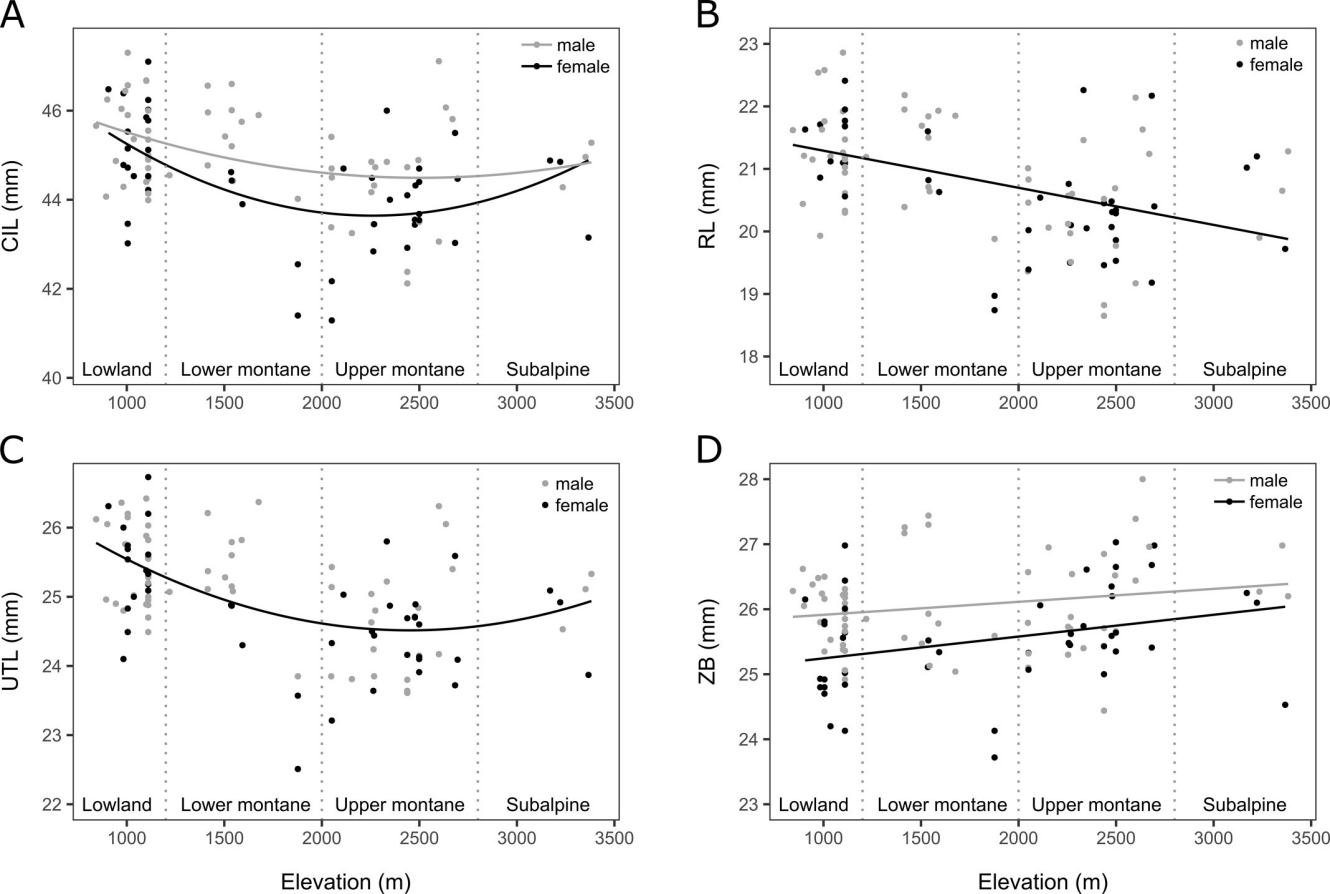
Elevational distribution of craniodental variation. Distribution of skull measurements taken from D6 individuals across the studied elevational gradient. Vegetation zonation is indicated with vertical dotted lines. (A) Condyle-incisive length (CIL), (B) rostrum length (RL), (C) upper tooth-row length (UTL), and (D) zygomatic breadth (ZB) across elevation. The limits of the vegetation zones are marked with vertical dotted lines. Black dots: females; gray dots: males. Lines show the relationship of the dependent variable with elevation for both sexes (only one line in the plot), for females (black lines) and for males (gray lines). (A) CIL followed a significant U-shaped distribution along elevation (*P* = 0.0038). Females showed significantly smaller CIL values than males (*P* = 0.0096) (CIL model *R*^*2*^_*adj*_ = 0.2363, *F*_3,102_ = 11.83). (B) RL significantly decreased linearly with elevation (*P* = 0.0148). No significant differences were found between sexes (RL model *R*^*2*^_*adj*_ = 0.8127, *F*_3,94_ = 141.3). (C) UTL followed a significant U-shaped distribution along elevation (*P* = 0.0378). No significant differences were found between sexes, but differences among mountains were significantly supported (*P* = 0.0167) (UTL model *R*^*2*^_*adj*_ = 0.818, *F*_6,98_ = 78.93). (D) ZB significantly increased linearly with elevation (*P* = 0.0004). Females showed significantly lower ZB values than males (*P* = 0.0079). ZB significantly increased over the years (*P* = 0.0177) (ZB model *R*^*2*^_*adj*_ = 0.3857, *F*_4,101_ = 17.48). CIL had a significant effect over RL (*P*< 0.00001), UTL (*P*< 0.00001) and ZB (*P*< 0.00001).

W and CIL followed a significant U-shaped distribution along elevation (D1; W: *F*_1,79_ = 4.6719, *P* = 0.0337; D6; CIL: *F*_1,102_ = 8.7565, *P* = 0.0038), (adjusted R^2^ (*R*^*2*^_*adj*_) and *F*-statistic for the models of W and CIL: W *R*^*2*^_*adj*_ = 0.2498, *F*_4,79_ = 7.909; CIL *R*^*2*^_*adj*_ = 0.2363, *F*_3,102_ = 11.83). This trend of W was also observed when analyzing D4 (**S1 File**). The smallest average values of W per vegetation zone were observed in the upper montane zone (mean = 123 g, standard error (*SE*) = 2.14), and in the lower montane zone (mean = 129 g, SE = 3.55) while W values were higher on average in the lowland zone (mean = 136, SE = 5.02) and in the subalpine zone (mean = 139, SE = 8.24). HBL did not show a significant trend over elevation in the small dataset analysis (D2; *F*_1,48_ = 0.1862, *P* = 0.6680), but did follow a significant U-shaped distribution along elevation in D4 analysis (**S1 File**) (HBL model *R*^*2*^_*adj*_ = -0.0326, *F*_4,79_ = 7.909) (**Figs 2** and **3A**).

Females showed significantly smaller CIL values than males (*F*_3,102_ = 6.9790, *P* = 0.0096; female mean CIL = 44.1 mm, *SE* = 0.20, sample size *N* = 45; male mean CIL = 44.6 mm, *SE* = 0.18, *N* = 61), while no significant differences between sexes were found for W or HBL (W: *F*_1,79_ = 0.0469, *P* = 0.8292; HBL: *F*_1,48_ = 0.1496, *P* = 0.7006). Similar results were obtained regarding sexual dimorphism when analyzing D4 (**S1 File**).

W followed a significant trend across years, with the most recently collected specimens increasing in W (*F*_1,79_ = 17.3116, *P*<0.0001); this result was also observed in the larger D4 dataset. HBL also significantly increased across years in the larger D4 dataset (**S1 File**). CIL did not change significantly over the years.

Differences among mountains in body size proxies were unsupported for the smaller datasets (D1 and D2), but these differences were statistically significant for W and HBL in the larger D4 analyses (**S1 File**).

### Allen’s rule

**Fig 4:**
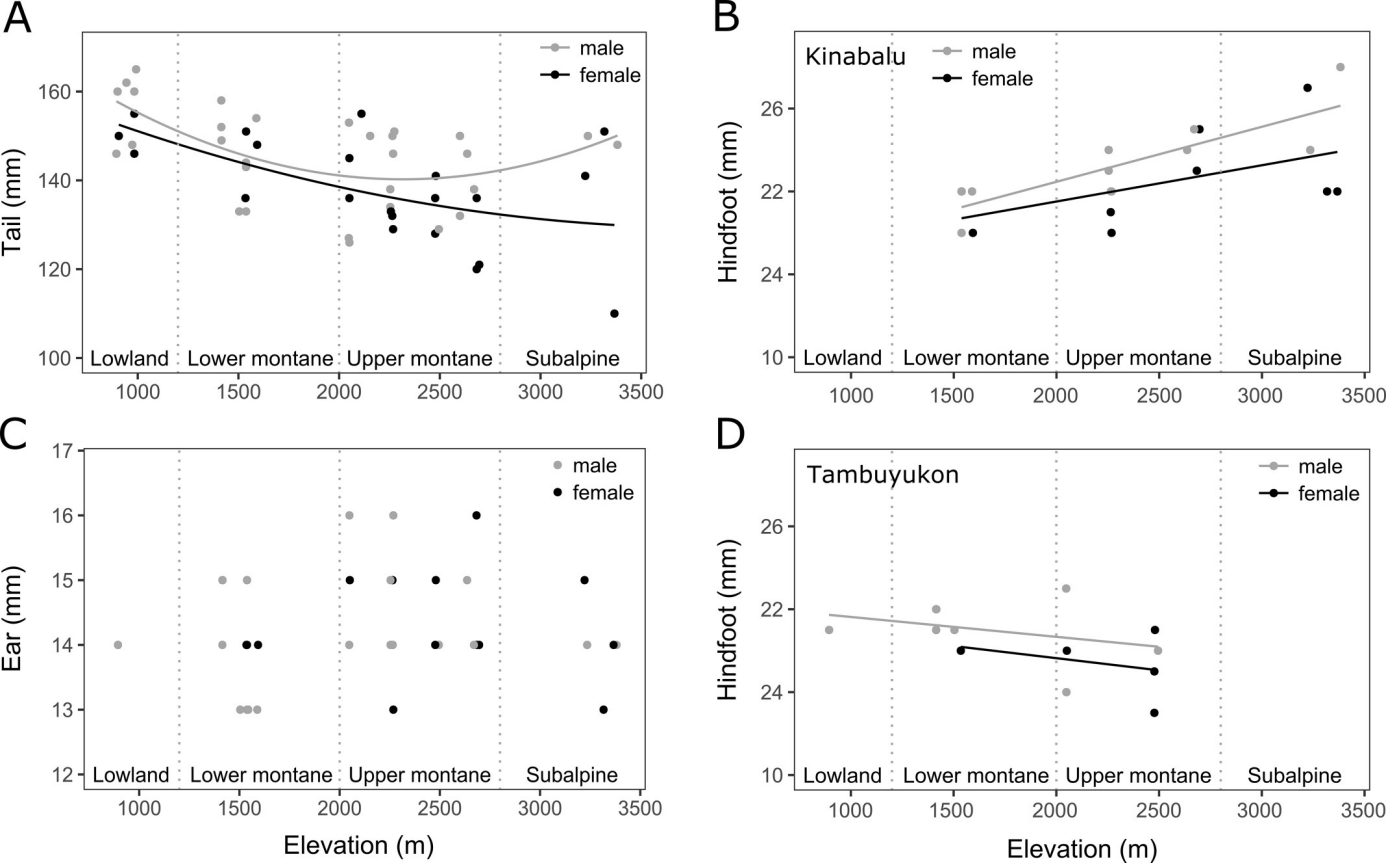
Elevational distribution of body shape. Distribution of (A) tail length (TL) of D2 individuals, (B and D) hindfoot length (HFL) of D3 individuals, and (C) ear length (EL) of D3 individuals (in mm) over the elevational gradient studied. Vegetation zonation indicated with vertical dotted lines. Black dots: females; gray dots: males. Lines show the relationship of the dependent variable with elevation for females (black lines) and for males (gray lines). (A) Tail followed a U-shaped distribution over elevation (*P =* 0.0163). Females had significantly shorter tails than males (*P* = 0.0321) (TL model *R*^*2*^_*adj*_ = 0.353, *F*_4.46_ = 7.821). (B and D) Although HFL globally significantly increased linearly over elevation (*P* = 0.0011), significant differences were observed between Mt. Kinabalu (B, N = 22) and Mt. Tambuyukon (D, N = 13, *P* = 0.0022). Females had significantly shorter hindfeet than males (HFL model *R*^*2*^_*adj*_ = 0.5665, *F*_4,30_ = 12.11). (C) No significant trends were observed for EL. HBL had a significant effect only over HFL (*P* = 0.0018).

The relative size of the appendages followed different patterns along elevation (**Fig 4**). TL followed a significant U-shaped distribution (*F*_1,46_ = 6.2188, *P =* 0.0163, TL model *R*^*2*^_*adj*_ = 0.353, *F*_4.46_ = 7.821), although males followed a linear decrease (**Fig 4A**). This was confirmed by the larger dataset D4, where a significant decrease in TL over elevation was observed (**S1 File**). EL did not follow a significant trend with elevation (**Fig 4C**, *F*_1,30_ = 0.0806, *P =* 0.7784, EL model *R*^*2*^_*adj*_ = -0.0895, *F*_3,30_ = 0.0966; D4, **S1 File**). HFL globally significantly increased linearly with elevation (**Fig 4B**, *F*_1,30_ = 12.954, *P* = 0.0011, HFL model *R*^*2*^_*adj*_ = 0.5665, *F*_4,30_ = 12.11), but showed significant differences between Mt. Kinabalu and Mt. Tambuyukon (*F*_1,30_ = 11.261, *P* = 0.0022): Mt. Kinabalu individuals had longer HFL (mean HFL = 44.5 mm, SE = 0.33, N = 22) than Mt. Tambuyukon ones (mean HFL = 42.6 mm, SE = 0.44, N = 13; HFL means averaged over sexes), and followed opposite trends along elevation.

Sexual dimorphism was statistically supported in TL (*F*_1,46_ = 4.8827, *P* = 0.0321) and HFL (*F*_1,30_ = 7.639, *P* = 0.0097). Females had shorter tails and hindfeet than males (female mean TL = 136 mm, SE = 2.49, N = 21; male mean TL = 142 mm, SE = 2.12, N = 30; female mean HFL = 42.8 mm, *SE* = 0.40, *N* = 15; male mean HFL = 44.3 mm, *SE* = 0.35, *N* = 20; HFL was averaged across mountains). No significant differences between sexes were observed for EL (*F*_1,30_ = 0.0670, *P* = 0.7975). No significant differences in EL and TL were found between mountains, although differences in TL among mountains were shown in D4 analysis (**S1 File**).

HBL showed a significant effect over HFL (*F*_1,30_ = 11.669, *P* = 0.0018) but not over EL (*F*_1,30_ = 0.1985, *P* = 0.6591) or TL (*F*_1,46_ = 3.1997, *P* = 0.0802). When analyzing the more extensive datasets D4 and D5, HBL had a significant effect over HFL, EL, and TL (**S1 File**).

### Diet-associated and insulation traits

Diet-related craniodental variables were distributed differently along elevation (**Fig 3C**, **3D**). While RL decreased linearly with elevation (*F*_1,94_ = 6.1665, *P* = 0.0148), UTL followed a U-shaped pattern (*F*_1,98_ = 4.4334, *P* = 0.0378), and ZB increased linearly (*F*_1,101_ = 13.6604, *P* = 0.0004) (RL model *R*^*2*^_*adj*_ = 0.8127, *F*_3,94_ = 141.3; UTL model *R*^*2*^_*adj*_ = 0.818, *F*_6,98_ = 78.93; ZB model *R*^*2*^_*adj*_ = 0.3857, *F*_4,101_ = 17.48). The three diet-related craniodental variables were significantly affected by CIL (RL *F*_1,94_ = 314.0722, *P*< 0.00001; UTL *F*_1,98_ = 271.1278, *P*< 0.00001; ZB *F*_1,94_ = 33.5914, *P*< 0.00001).

Significant sexual dimorphism was observed in ZB (*F*_1,101_ = 7.3389, *P* = 0.0079; female mean ZB = 25.6 mm, *SE* = 0.09, *N* = 45; male mean ZB = 26.0 mm, *SE* = 0.08, *N* = 61). As with HFL, the small difference among sexes is unlikely to be biologically significant. Significant differences were observed among mountains only in UTL (*F*_2,98_ = 4.2664, *P* = 0.0167), with a significantly smaller UTL on Mt. Kinabalu than on Mt. Tambuyukon (*posthoc Tukey test estimate* = -0.23, *df* = 98, *P* = 0.0275; Mt. Kinabalu, mean UTL = 24.85 mm, SE = 0.06, N = 77; Mt. Tambuyukon, mean UTL = 25.08 mm, SE = 0.07, N = 23; Mt. Trus Madi, mean UTL = 25.13 mm, SE = 0.16, N = 5, UTL means averaged over sexes). A significant trend across years was observed only for ZB, with higher values in the more recent individuals (*F*_1,101_ = 5.8175, *P* = 0.0177).

SMI did not significantly change over elevation (*F*_1,45_ = 1.2575, *P* = 0.2681) or between sexes (*F*_1,45_ = 0.0856, *P* = 0.7712) (**Fig 3 in S1 File**). No significant differences were observed among mountains (*F*_2,45_ = 0.8265, *P* = 0.4441) or years (*F1*_,45_ = 1.1595, *P* = 0.2873) (model *R*^*2*^_*adj*_ = -0.0190; model *F*_5,45_ = 0.8132) (**S1 File**).

**Fig 5:**
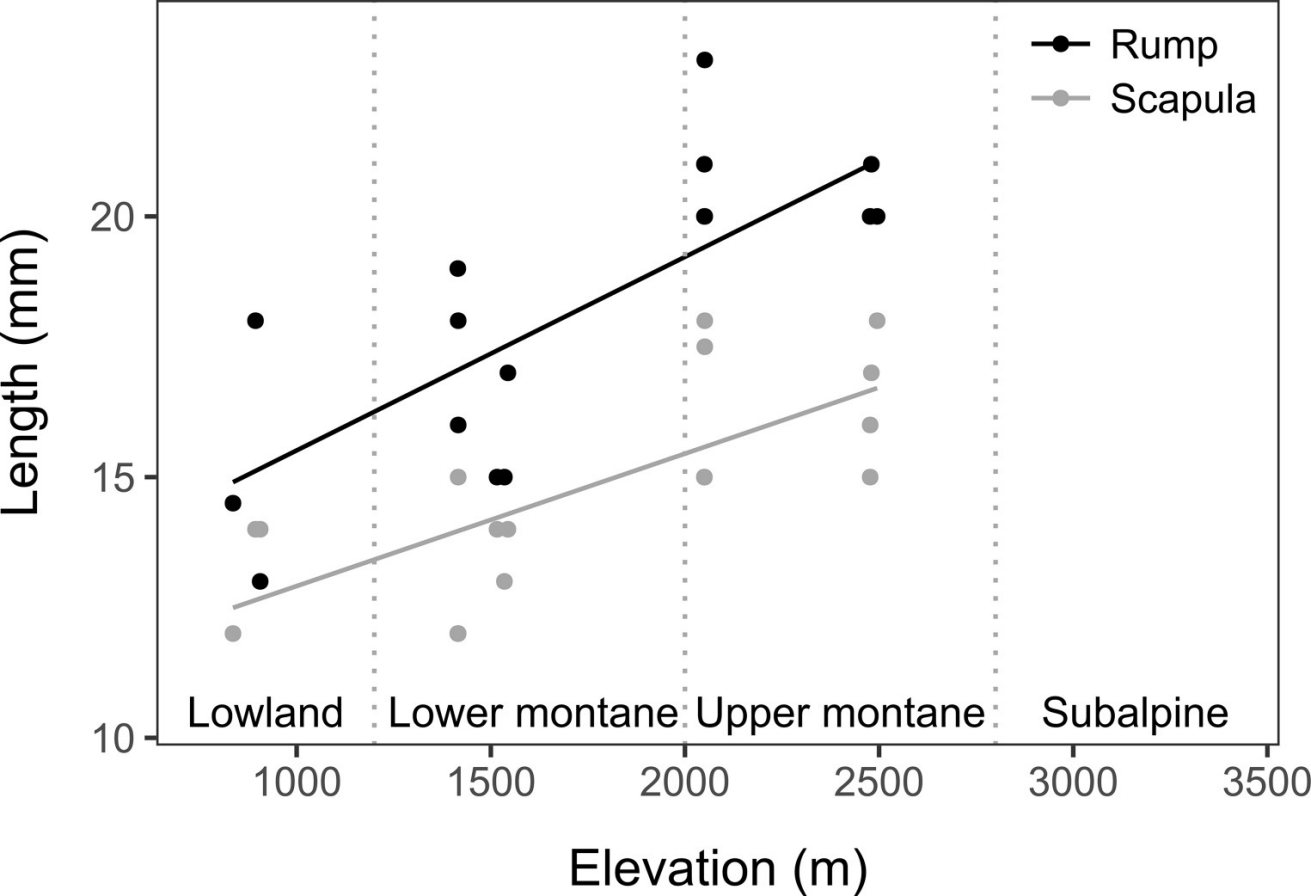
Elevational distribution of dorsum hair length. Distribution of dorsum hair length (in mm) at the rump (HRL) and scapula (HSL) of Mountain Treeshrews over an elevational gradient (in m) on Mt. Tambuyukon. The limits of the vegetation zones are marked with vertical dotted lines. Hair length (HRL, HSL) significantly increased with elevation (HRL model *R*^*2*^_*adj*_ = 0.5618, *F*_1,15_ = 21.52, *P* = 0.0003; HSL model *R*^*2*^_*adj*_ = 0.5033, *F*_1,15_ = 17.21, *P* = 0.0009).

Hair length (HRL, HSL) significantly increased with elevation (*R*^*2*^_*adj*_ = 0.5618, *F*_1,15_ = 21.52, *P* = 0.0003; *R*^*2*^_*adj*_ = 0.5033, *F*_1,15_ = 17.21, *P* = 0.0009, **Fig 5**).

## Discussion

Bergmann’s rule was not supported in our tropical, montane (elevation) system. Skull length, head-and-body length, and weight decreased from the lowest elevations (<1000 m) to middle elevations (2000–2500 m), then increased from middle to higher elevations, showing a pattern different from that predicted by this rule. This is in contrast with sympatric Bornean shrews which increase with size along elevation [55], but mirrors other studies along tropical elevational gradients such as the New Guinean rats [31], Neotropical Soft Grass mice [32], Wood-wrens [56], and Torrent ducks [36] or subtropical small mammals [6] which were also inconsistent with this rule, with individuals generally becoming smaller with elevation. Neotropical Howler monkeys, Water and Woolly opossums, peccaries, and Crab-eating Fox also followed an inverse Bergmann’s rule pattern across latitude at an interspecific or intraspecific level, although the Crab-eating Fox also showed a strong Bergmannian pattern south of the Equator [33, 57–60].

In the Mountain Treeshrew, individuals from the upper montane forest (2000–2500 m elevation range) exhibited the smallest size and weight. A similar trend was shown for the Yunnan Field Mouse (*Apodemus ilex*) in Southern China, which decreased in size with elevation up to 3200 m and then exhibited a strong positive relationship with elevation [6]. The previously documented negative correlation between temperature and primary productivity with elevation found in Mt. Kinabalu [42], suggests alternative selective forces may drive these phenotypic changes. Different environmental or biotic factors such as increasing rainfall and water surplus, reduced visibility generated from thick mist, lowered nutrient availability, decreasing radiation, changes in food quality/abundance, and increasing intraspecific competition (the highest population density is found in the upper montane forest) might be interacting and shaping this mid-elevation body size decrease (see [61] for a scheme on the complex interactions that can govern body size in island mammals). Similarly, the unexpected increase in size in the open stunted forest and subalpine meadows of Mt. Kinabalu could be due to: a predator release effect, a reduction of interspecific competition (since Mountain Treeshrews share that habitat with just five other small mammal species [39]), or an increase in relevant food resources. In any case, testing these alternative mechanistic hypotheses is beyond the scope of this study and will require a combination of additional ecological, physiological, and diet data.

In this study system, only relative tail length was consistent with Allen’s rule, which decreased with elevation (in the larger D4 and females of D2), as the rule predicts. Ear and hindfoot length did not follow this rule, since the former remained constant, and the latter increased with elevation (although differences were found among mountains). Allen’s rule has previously been supported for relative tail length in Xeric Four-striped Grass Rat (*Rhabdomys pumilio*) across elevation; and Virginia opossums (*Didelphis virginiana*), Herb Field mice (*Apodemus uralensis*), and Long-tailed macaques (*Macaca fascicularis*) across latitude; but not for South African Mouse shrews (*Myosorex varius*) or Soft-furred Tree mice (*Typhlomys cinereus*) across elevation, or fossorial Southern African Pouched mice (*Saccostomus campestris)* across latitude [3, 7, 26, 62–64]. Interestingly, tail length seems to exhibit a stronger relationship than ear or hindfoot length with elevation [3] and latitude [65, 66]. The factors driving this stronger relationship might be biological (e.g. temperature) or non-biological ones such as those derived from lower measurement errors from the larger appendages (i.e. tail) compared to the smallest (i.e. ear and hindfoot) when instruments with the same precision are used. In fact, [65] found that temperature poorly predicted tail length (or other extremities) and hypothesized that hindfoot length likely relates to arboreality in the tropics. Longer tails enhance aerodynamic performance and greater maneuverability, which are important to escape predation by leaping, and to improve foraging [65, 67]. Tail length has been shown to be related to scansoriality in squirrels [67] and cricetids [68], but it has only been hypothesized (but not tested) in treeshrews [69]. In the oak-dominated (Fagaceae) upper montane forest of Borneo the main resources provided in the canopy might be hard shell nuts, which treeshrews cannot feed on [40, 41]. This could perhaps encourage more terrestrial foraging at higher elevations, mirroring the pattern shown along latitude in New World small mammals [65]. Thus, the trend observed in treeshrew tail length might be the result of two converging selective agents (temperature and locomotion).

The rule reversal pattern shown in hindfoot length along elevation could also be consistent with more terrestrial locomotion and foraging at higher elevations (longer hindfoot) and a more scansorial fruit foraging behavior at lower elevations (short hindfoot and longer balancing tail). However, most of this study’s Mountain Treeshrews were sampled in the ground, perhaps suggesting a predominantly terrestrial foraging with different degrees of scansoriality across elevation [39]. Similarly, highland bird communities of our study area were also shown to contain species with relatively longer tarsus than lowland relatives [35]. Relative rostrum length decreased significantly with elevation, but it visually followed the same U-shaped pattern with elevation that CIL (**Fig 3**) and was highly correlated with it, casting doubts on whether the trend is biologically significant or if it might be driven by allometric covariation. Similarly, UTR length followed the same pattern with elevation as CIL, so this trait might also be driven by allometric covariation. UTR is a developmentally constrained trait [70], so stasis is expected, particularly in treeshrews which are not considered dietary specialists [71]. Phenotypic stasis in rostrum length would contrast with other studies [17].

Zygomatic arches are where masseter muscles attach and are integral to the biomechanics of mastication. In Mountain Treeshrews the zygomatic breadth increases along elevation, which could indicate adaptation to a diet with harder food items [72–74]. The observed increase in zygomatic breadth parallels the decrease in availability of soft fleshy fruits and some invertebrates with elevation [41, 75]. Further diet studies will be required to address this hypothesis.

While body size changed along elevation, body condition, an important trait that can be considered a surrogate of lipid content and is characteristic of environmental stress [54], did not. This lack of body condition variation might suggest successful local adaptation throughout its elevational range, perhaps due to generalist foraging strategies [4].

Sexual size dimorphism in the Mountain Treeshrew was statistically significant for some traits (skull length, zygomatic breadth, tail and hindfoot length), which contrasts with other studies on different treeshrew species [41, 45]. The observation of morphological differentiation on a backdrop of high geneflow [76] further highlights the possibility that this pattern is caused by phenotypic plasticity or strong divergent selection, which are some of the mechanisms that could maintain local adaptation despite gene flow [77–81]. Similar patterns of phenotypic differentiation with shallow genetic divergence have been observed in mountain chickadees [82] and Eurasian tree sparrows [83]. Additional novel, functional genomic, transcriptomic and/ or epigenetic approaches will be needed to address the specific molecular basis of these phenotypic traits.

Overall body size may be related to compliance with Bergman’s and Allen’s rules. Several studies [8, 10] have found a significantly lower tendency to conform to Bergmann’s rule within smaller mammals, more specifically, those with a body mass under 500 g, which is the case of the Mountain Treeshrew. Small mammals show alternative strategies to overcome changes in temperature and resource availability than larger ones. Small mammals are better at exploiting torpor, food caching, and microclimatic refugia through burrowing or nesting [84]. Mountain Treeshrews are diurnal and can behaviorally thermoregulate with the use of burrows when necessary, which could reduce the selective pressure of temperature on them as compared to nocturnal species or larger species that are more exposed to rainy and cold weather. An additional mechanism that may mitigate the effect of temperature on body size in this system is conspicuous, possibly adaptive changes in insulation across elevation [85]. High-elevation individuals have softer, more wooly underfur, with a conspicuously higher hair density and longer hairs than lower elevation Mountain Treeshrews (**Fig 5**; **S1 File**), similar to that found in sympatric *Crocidura foetida sensu lato-C*. *baluensis* [55], *Peromyscus maniculatus* [19], and New Guinean *Rattus* sp. [31].

A highly significant temporal effect was detected in several traits (in D1, D4 and D5 datasets). A temporal trend of increasing weight and head-and-body length was supported, but the magnitude of such changes is difficult to quantify due to the effect of other interacting variables such as elevation. Similarly, ear length decreased and hindfoot length increased with time, while tail length and body condition did not. Given that external measurements are prone to observer bias we remain highly skeptical of any temporal trends since these have not been verified with a less error-prone dataset as in the elevation analyses [46, 47]. Furthermore, skull length, which is exempt of such associated errors, did not increase across years. This lack of body size change across the last century represents the first temporal body size trend described on treeshrews. It mirrors that of other species such as Cape hares and Golden jackals in Israel [86], California Ground squirrels [71], Smith’s Red-backed voles [87] and all but 6 of the 52 populations of 22 species of carnivores analyzed in [88], but is in contrast with other species such as Northern chamois [89] and African Wild dogs [90] which have been shown to decrease in size or Belding’s Ground squirrels and Common Golden-mantled Ground squirrels [68], Large Japanese Field mice [87], Masked shrews [91] and Spain and Israel’s Red foxes [86, 92] which have been shown to increase in size. In the light of the average temperature increase of the last century, this lack of temporal body size change is consistent with our elevational findings, which suggest that temperature is not an important force shaping the morphological variation of the Mountain Treeshrew. It should also be noted that most studies assessing temporal body size variation are biased toward higher latitudes, where there has been a greater temperature increase than in the tropics during the last century [93]. Additional studies will be required to address whether temporal body size changes are less common in the tropics. The absence of an observed change in body size for CIL may also be the result of low statistical power due to the smaller sample size for this trait than HBL and W rather than indicating tolerance of climatic changes [94]. Relative zygomatic breadth increased through the years, perhaps driven by vegetation zonation shifts and associated changes in resource availability. Future studies including newly collected and additional museum specimens will be required to address any of these temporal trends with higher confidence.

These data from a tropical system both support and refute these long-standing biogeographical rules in ways that might shed light on conflicting observations recorded in the literature. Small mammals may experience their environment in a fundamentally different way through their ability to modify their own microenvironment. There also may be essential differences in the environmental clines generated by latitude vs. elevation. One obvious difference is scale, which impacts population size and gene flow and thus the efficiency of selection. Variability in the environment, such as seasonality, also has a major impact on selection. Small mammals in more seasonal mountains might comply more with Bergmann’s rule since resource-shortage periods at higher elevations are longer in colder areas. An increase in size (and fat reserves) in these colder habitats might allow individuals to cope better with these periods. The trends shown in tropical New Guinean rats, Mountain Treeshrews, and subtropical Chinese small mammals suggest a lack of support for Bergmann’s rule in tropical/ subtropical mountain small mammals. Understanding morphological patterns across elevational gradients can provide important insights on how species adapt to changes in different environmental factors and can thereby shed light on how montane specialists could react under a climate change scenario. Overall, our study suggests that selective pressures other than temperature, perhaps driven by diet, food acquisition, predation pressure and/or intra- and inter-specific competition, are playing an important role shaping the morphological variation of the Mountain Treeshrew.

## Supporting information

S1 Table Table of specimens examined in this study, its associated information and morphological data. (XLSX)

S1 File Appendix. Methods, results and figures of dataset D2 statistical analyses, figure of body condition, and pictures highlighting differences in fur of Mountain Treeshrews (*Tupaia montana)* along elevation.
(DOCX)

S2 File Checklist. Additional information regarding the ethical, cultural, and scientific considerations specific to inclusivity in global research.
(PDF)

## References

[1] Bergmann C. Über die Verhältnisse der Wärmeökonomie der Thiere zu ihrer Grösse. 1848.

[2] Allen JA. The influence of physical conditions in the genesis of species. Radic Rev. 1877;1:108–40.

[3] Rowe-Rowe J DT* & Meester. Altitudinal variation in external measurements of two small-mammal species in the Natal Drakensberg. Ann Transvaal Mus. 1985;34(3):49–53.

[4] Keller I, Alexander JM, Holderegger R, Edwards PJ. Widespread phenotypic and genetic divergence along altitudinal gradients in animals. J Evol Biol. 2013;26(12):2527–43. doi: 10.1111/jeb.12255 24128377

[5] Du Y, Wen Z, Zhang J, Lv X, Cheng J, Ge D, et al. The roles of environment, space, and phylogeny in determining functional dispersion of rodents (Rodentia) in the Hengduan Mountains, China. Ecol Evol. 2017;7(24):10941–51. doi: 10.1002/ece3.3613 29299271 PMC5743695

[6] Feijo A, Wen Z, Cheng J, Ge D, Xia L, Yang Q. Divergent selection along elevational gradients promotes genetic and phenotypic disparities among small mammal populations. Ecol Evol. 2019;9(12):7080–95. doi: 10.1002/ece3.5273 31380035 PMC6662404

[7] Cui J, Lei B, Newman C, Ji S, Su H, Buesching CD, et al. Functional adaptation rather than ecogeographical rules determine body-size metrics along a thermal cline with elevation in the Chinese pygmy dormouse (*Typhlomys cinereus*). J Therm Biol. 2020;88:102510. doi: 10.1016/j.jtherbio.2020.102510 32125991

[8] Ashton KG, Tracy MC, Queiroz A de. Is Bergmann’s rule valid for mammals? Am Nat. 2000;156(4):390–415. doi: 10.1086/303400 29592141

[9] Freckleton RP, Harvey PH, Pagel M. Bergmann’s rule and body size in mammals. Am Nat. 2003;161(5):821–5. doi: 10.1086/374346 12858287

[10] Meiri S, Dayan T. On the validity of Bergmann’s rule. J Biogeogr. 2003;30(3):331–51.

[11] Yom-Tov Y, Geffen E. Geographic variation in body size: the effects of ambient temperature and precipitation. Oecologia. 2006;148(2):213–8. doi: 10.1007/s00442-006-0364-9 16525785

[12] Alhajeri BH, Steppan SJ. Association between climate and body size in rodents: a phylogenetic test of Bergmann’s rule. Mamm Biol. 2016;81(2):219–25.

[13] Vega R, Mcdevitt AD, Kryštufek B, Searle JB. Ecogeographical patterns of morphological variation in pygmy shrews *Sorex minutus* (Soricomorpha: Soricinae) within a phylogeographical and continental-and-island framework. Biol J Linn Soc. 2016;119(4):799–815.

[14] Brown JH, Lee AK. Bergmann’s rule and climatic adaptation in woodrats (*Neotoma*). Evolution. 1969;329–38. doi: 10.1111/j.1558-5646.1969.tb03515.x 28562890

[15] Ralls K, Harvey PH. Geographic variation in size and sexual dimorphism of North American weasels. Biol J Linn Soc. 1985;25(2):119–67.

[16] Yom-Tov Y, Nix H. Climatological correlates for body size of five species of Australian mammals. Biol J Linn Soc. 1986;29(4):245–62.

[17] Lindsay SL. Geographic size and non-size variation in Rocky Mountain *Tamiasciurus hudsonicus*: Significance in relation to Allen’s rule and vicariant biogeography. J Mammal. 1987;68(1):39–48.

[18] Gür H. Why do Anatolian ground squirrels exhibit a Bergmannian size pattern? A phylogenetic comparative analysis of geographic variation in body size. Biol J Linn Soc. 2010;100(3):695–710.

[19] Wasserman D, Nash DJ. Variation in body size, hair length, and hair density in the deer mouse *Peromyscus maniculatus* along an altitudinal gradient. Ecography. 1979;2(2):115–8.

[20] Liao J, Zhang Z, Liu N. Altitudinal variation of skull size in Daurian pika (*Ochotona daurica* Pallas, 1868). Acta Zool Acad Sci Hung. 2006;52(3):319–29.

[21] Bidau CJ, Martí DA, Medina AI. A test of Allen’s rule in subterranean mammals: the genus *Ctenomys* (Caviomorpha, Ctenomyidae). 2011.

[22] Müller J, Bässler C, Essbauer S, Schex S, Müller DWH, Opgenoorth L, et al. Relative heart size in two rodent species increases with elevation: reviving Hesse’s rule. J Biogeogr. 2014;41(12):2211–20.

[23] Sargis EJ, Millien V, Woodman N, Olson LE. Rule reversal: ecogeographical patterns of body size variation in the common treeshrew (Mammalia, Scandentia). Ecol Evol. 2018;8(3):1634–45. doi: 10.1002/ece3.3682 29435239 PMC5792578

[24] Stanchak KE, Santana SE. Do ecogeographical rules explain morphological variation in a diverse, Holarctic genus of small mammals? J Biogeogr. 2019;46(1):110–22.

[25] Ochocińska D, Taylor JR. Bergmann’s rule in shrews: geographical variation of body size in Palearctic *Sorex* species. Biol J Linn Soc. 2003;78(3):365–81.

[26] Balčiauskas L, Amshokova A, Balčiauskienė L, Benedek AM, Cichocki J, Csanady A, et al. Geographical clines in the size of the herb field mouse (*Apodemus uralensis*). Integr Zool. 2020;15(1):55–68. doi: 10.1111/1749-4877.12407 31149780

[27] Boyce MS. Seasonality and patterns of natural selection for life histories. Am Nat. 1979;114(4):569–83.

[28] Murphy EC. Bergmann’s rule, seasonality, and geographic variation in body size of house sparrows. Evolution. 1985;39(6):1327–34. doi: 10.1111/j.1558-5646.1985.tb05698.x 28564272

[29] Zeveloff SI, Boyce MS, others. Body size patterns in North American mammal faunas. Evol Life Hist Mamm. 1988;123–46.

[30] Chown SL, Klok CJ. Altitudinal body size clines: latitudinal effects associated with changing seasonality. Ecography. 2003;26(4):445–55.

[31] Taylor JM, Smith SC, Calaby JH. Altitudinal distribution and body size among New Guinean *Rattus* (Rodentia: Muridae). J Mammal. 1985;66(2):353–8.

[32] Alvarado-Serrano DF, Luna L, Knowles LL. Localized versus generalist phenotypes in a broadly distributed tropical mammal: how is intraspecific variation distributed across disparate environments? BMC Evol Biol. 2013;13(1):1–16. 23899319 10.1186/1471-2148-13-160 PMC3737017

[33] Hendges CD, Patterson BD, Cáceres NC. Big in the tropics: Ecogeographical clines in peccary size reveal the converse of Bergmann’s rule. J Biogeogr. 2021;48(5):1228–39.

[34] Freeman BG. Little evidence for Bergmann’s rule body size clines in passerines along tropical elevational gradients. J Biogeogr. 2017;44(3):502–10.

[35] Boyce AJ, Shakya S, Sheldon FH, Moyle RG, Martin TE. Biotic interactions are the dominant drivers of phylogenetic and functional structure in bird communities along a tropical elevational gradient. The Auk. 2019;136(4):ukz054.

[36] Gutiérrez-Pinto N, McCracken KG, Alza L, Tubaro P, Kopuchian C, Astie A, et al. The validity of ecogeographical rules is context-dependent: testing for Bergmann’s and Allen’s rules by latitude and elevation in a widespread Andean duck. Biol J Linn Soc. 2014;111(4):850–62.

[37] Morgan K, Mboumba J-F, Ntie S, Mickala P, Miller CA, Zhen Y, et al. Precipitation and vegetation shape patterns of genomic and craniometric variation in the Central African rodent *Praomys misonnei*. Proc R Soc B. 2020;287(1930):20200449. doi: 10.1098/rspb.2020.0449 32635865 PMC7423474

[38] Stevens GC. The latitudinal gradient in geographical range: how so many species coexist in the tropics. Am Nat. 1989;133(2):240–56.

[39] Camacho-Sanchez M, Hawkins MT, Yu FTY, Maldonado JE, Leonard JA. Endemism and diversity of small mammals along two neighboring Bornean mountains. PeerJ. 2019; 7: e7858. doi: 10.7717/peerj.7858 31608182 PMC6788440

[40] Lim B, Heyneman D. A collection of small mammals from Tuaran and the southwest face of Mt Kinabalu, Sabah. Sarawak Mus J. 1968;16:257–78.

[41] Emmons LH. Tupai: A Field Study of Bornean Treeshrews [Internet]. Tupai. University of California Press; 2000 [cited 2021 Oct 2]. Available from: https://www.degruyter.com/document/doi/10.1525/9780520925045/html.

[42] Kitayama K. An altitudinal transect study of the vegetation on Mount Kinabalu, Borneo. Vegetatio. 1992;102(2):149–71.

[43] Kitayama K, Kulip J, Nais J, Biun A. Vegetation survey on Mount Trus Madi, Borneo a prospective new Mountain Park. Mt Res Dev. 1993;13(1):99–105.

[44] Sikes RS, Care A, Mammalogists UC of the AS of. 2016 Guidelines of the American Society of Mammalogists for the use of wild mammals in research and education. J Mammal. 2016;97(3):663–88. doi: 10.1093/jmammal/gyw078 29692469 PMC5909806

[45] Woodman N, Miller-Murthy A, Olson LE, Sargis EJ. Coming of age: morphometric variation in the hand skeletons of juvenile and adult Lesser Treeshrews (Scandentia: Tupaiidae: *Tupaia minor* Günther, 1876). J Mammal. 2020;101(4):1151–64.

[46] Blackwell GL, Bassett SM, Dickman CR. Measurement error associated with external measurements commonly used in small-mammal studies. J Mammal. 2006;87(2):216–23.

[47] Stephens RB, Karau KH, Yahnke CJ, Wendt SR, Rowe RJ. Dead mice can grow–variation of standard external mammal measurements from live and three postmortem body states. J Mammal. 2015;96(1):185–93.

[48] Musser GG, Durden LA, Holden ME, Light JE. Systematic review of endemic Sulawesi squirrels (Rodentia, Sciuridae), with descriptions of new species of associated sucking lice (Insecta, Anoplura), and phylogenetic and zoogeographic assessments of sciurid lice. Bull Am Mus Nat Hist. 2010;2010(339):1–260.

[49] Fox J, Weisberg S. An R companion to applied regression. Sage publications; 2018.

[50] Lenth R, Singmann H, Love J, Buerkner P, Herve M. Emmeans: Estimated marginal means, aka least-squares means. R Package Version. 2018;1(1):3.

[51] Team RC, others. R: A language and environment for statistical computing. 2013.

[52] Allaire J. RStudio: integrated development environment for R. Boston MA. 2012;770(394):165–71.

[53] Peig J, Green AJ. New perspectives for estimating body condition from mass/length data: the scaled mass index as an alternative method. Oikos. 2009;118(12):1883–91.

[54] Peig J, Green AJ. The paradigm of body condition: a critical reappraisal of current methods based on mass and length. Funct Ecol. 2010;24(6):1323–32.

[55] Hinckley A, Camacho-Sanchez M, Ruedi M, Hawkins MTR, Mullon M, Cornellas A, et al. Evolutionary history of Sundaland shrews (Eulipotyphla: Soricidae: *Crocidura*) with a focus on Borneo. Zool J Linn Soc [Internet]. 2021 Jul 24 [cited 2021 Oct 18]; (zlab045). Available from: 10.1093/zoolinnean/zlab045.

[56] Caro LM, Caycedo-Rosales PC, Bowie RCK, Slabbekoorn H, Cadena CD. Ecological speciation along an elevational gradient in a tropical passerine bird? J Evol Biol. 2013;26(2):357–74. doi: 10.1111/jeb.12055 23298144

[57] Martinez PA, Marti DA, Molina WF, Bidau CJ. Bergmann’s rule across the equator: a case study in *Cerdocyon thous* (Canidae). J Anim Ecol. 2013;82(5):997–1008. doi: 10.1111/1365-2656.12076 23550718

[58] Cerqueira R, Weber MM. Geographic morphometric and environmental differentiation of the water opossum, genus *Chironectes* Illiger, 1811 (Didelphimorphia: Didelphidae). Mammalia. 2017;81(3):275–87.

[59] Magnus LZ, Machado RF, Cáceres N. Comparative ecogeographical variation in skull size and shape of two species of woolly opossums (genus *Caluromys*). Zool Anz. 2017;267:139–50.

[60] Bubadué J, Polidoro G, Melo G, Sponchiado J, Serio C, Melchionna M, et al. Rensch’s and Bergmann’s Rules in Cis-Andean South-American Howler Monkeys (Mammalia: *Alouatta*). Hystrix Ital J Mammal. 2018.

[61] Heaney LR. Island Area and Body Size of Insular Mammals: Evidence from the Tri-Colored Squirrel (*Callosciurus prevosti*) of Southeast Asia. Evolution. 1978;32(1):29–44. doi: 10.1111/j.1558-5646.1978.tb01096.x 28564084

[62] Ellison GTH, Taylor PJ, Nix HA, Bronner GN, McMahon JP. Climatic Adaptation of Body Size Among Pouched Mice (*Saccostomus campestris*: Cricetidae) in the Southern African Subregion. Glob Ecol Biogeogr Lett. 1993 Mar;3(2):41.

[63] Fooden J, Albrecht GH. Tail-length evolution in fascicularis-group macaques (Cercopithecidae: *Macaca*). Int J Primatol. 1999;20(3):431–40.

[64] Nigenda-Morales SF, Harrigan RJ, Wayne RK. Playing by the rules? Phenotypic adaptation to temperate environments in an American marsupial. PeerJ. 2018;6: e4512. doi: 10.7717/peerj.4512 29607255 PMC5877449

[65] Alroy J. Small mammals have big tails in the tropics. Glob Ecol Biogeogr. 2019;28(8):1042–50.

[66] Alhajeri BH, Fourcade Y, Upham NS, Alhaddad H. A global test of Allen’s rule in rodents. Glob Ecol Biogeogr. 2020;29(12):2248–60.

[67] Hayssen V. Patterns of body and tail length and body mass in Sciuridae. J Mammal. 2008;89(4):852–73.

[68] Kingsley EP, Kozak KM, Pfeifer SP, Yang D-S, Hoekstra HE. The ultimate and proximate mechanisms driving the evolution of long tails in forest deer mice. Evolution. 2017;71(2):261–73. doi: 10.1111/evo.13150 27958661 PMC5324611

[69] Martin R. Reproduction and ontogeny in tree-shrews (*Tupaia belangeri*), with reference to their general behaviour and taxonomic relationships. Z Für Tierpsychol. 1968;25(5):505–32. 10.1111/j.1439-0310.1968.tb00026.x 5710022

[70] Lessa EP, Patton JL. Structural constraints, recurrent shapes, and allometry in pocket gophers (genus *Thomomys*). Biol J Linn Soc. 1989;36(4):349–63.

[71] Eastman LM, Morelli TL, Rowe KC, Conroy CJ, Moritz C. Size increase in high elevation ground squirrels over the last century. Glob Change Biol. 2012;18(5):1499–508.

[72] Monteiro LR, Duarte LC, dos Reis SF. Environmental correlates of geographical variation in skull and mandible shape of the punaré rat *Thrichomys apereoides* (Rodentia: Echimyidae). J Zool. 2003;261(1):47–57.

[73] Samuels JX. Cranial morphology and dietary habits of rodents. Zool J Linn Soc. 2009;156(4):864–88.

[74] Hautier L, Lebrun R, Cox PG. Patterns of covariation in the masticatory apparatus of hystricognathous rodents: implications for evolution and diversification. J Morphol. 2012;273(12):1319–37. doi: 10.1002/jmor.20061 22833466

[75] Brühl CA, Mohamed M, Linsenmair KE. Altitudinal distribution of leaf litter ants along a transect in primary forests on Mount Kinabalu, Sabah, Malaysia. J Trop Ecol. 1999;15(3):265–77.

[76] Parker LD, Hawkins MT, Camacho-Sanchez M, Campana MG, West-Roberts JA, Wilbert TR, et al. Little genetic structure in a Bornean endemic small mammal across a steep ecological gradient. Mol Ecol. 2020;29(21):4074–90. doi: 10.1111/mec.15626 32911576

[77] Cheviron ZA, Connaty AD, McClelland GB, Storz JF. Functional genomics of adaptation to hypoxic cold-stress in high-altitude deer mice: transcriptomic plasticity and thermogenic performance. Evolution. 2014;68(1):48–62. doi: 10.1111/evo.12257 24102503 PMC4399701

[78] Tigano A, Friesen VL. Genomics of local adaptation with gene flow. Mol Ecol. 2016;25(10):2144–64. doi: 10.1111/mec.13606 26946320

[79] Rodríguez A, Rusciano T, Hamilton R, Holmes L, Jordan D, Wollenberg Valero KC. Genomic and phenotypic signatures of climate adaptation in an *Anolis* lizard. Ecol Evol. 2017;7(16):6390–403. doi: 10.1002/ece3.2985 28861242 PMC5574798

[80] Schmid M, Guillaume F. The role of phenotypic plasticity on population differentiation. Heredity. 2017;119(4):214–25. doi: 10.1038/hdy.2017.36 28745716 PMC5597782

[81] Montero-Mendieta S, Tan K, Christmas MJ, Olsson A, Vilà C, Wallberg A, et al. The genomic basis of adaptation to high-altitude habitats in the eastern honey bee (*Apis cerana*). Mol Ecol. 2019;28(4):746–60. doi: 10.1111/mec.14986 30576015

[82] Branch CL, Jahner JP, Kozlovsky DY, Parchman TL, Pravosudov VV. Absence of population structure across elevational gradients despite large phenotypic variation in mountain chickadees (*Poecile gambeli*). R Soc Open Sci. 2017;4(3):170057. doi: 10.1098/rsos.170057 28405402 PMC5383859

[83] Qu Y, Chen C, Xiong Y, She H, Zhang YE, Cheng Y, et al. Rapid phenotypic evolution with shallow genomic differentiation during early stages of high elevation adaptation in Eurasian Tree Sparrows. Natl Sci Rev. 2020;7(1):113–27. doi: 10.1093/nsr/nwz138 34692022 PMC8289047

[84] Blackburn TM, Gaston KJ, Loder N. Geographic gradients in body size: a clarification of Bergmann’s rule. Divers Distrib. 1999;5(4):165–74.

[85] Scholander PF. Evolution of climatic adaptation in homeotherms. Evolution. 1955;15–26.

[86] Yom-Tov Y, Yom-Tov S. Observations on variation in skull size of three mammals in Israel during the 20th century. Zool Anz—J Comp Zool. 2012 Nov 1;251(4):331–4.

[87] Yom-Tov Y, Yom-Tov S. Climatic change and body size in two species of Japanese rodents. Biol J Linn Soc. 2003 Sep 1;82(2):263–7.

[88] Meiri S, Guy D, Dayan T, Simberloff D. Global change and carnivore body size: data are stasis. Glob Ecol Biogeogr. 2009;18(2):240–7.

[89] Mason TH, Apollonio M, Chirichella R, Willis SG, Stephens PA. Environmental change and long-term body mass declines in an alpine mammal. Front Zool. 2014 Sep 27;11(1):69.

[90] Mcnutt JW, Gusset M. Declining body size in an endangered large mammal. Biol J Linn Soc. 2012 Jan 1;105(1):8–12.

[91] Yom-Tov Y, Yom-Tov J. Global warming, Bergmann’s rule and body size in the masked shrew Sorex cinereus Kerr in Alaska. J Anim Ecol. 2005;74(5):803–8.

[92] Yom-Tov Y, Yom-Tov S, Barreiro J, Blanco JC. Body size of the red fox *Vulpes vulpes* in Spain: the effect of agriculture. Biol J Linn Soc. 2007 Apr 1;90(4):729–34.

[93] Deutsch CA, Tewksbury JJ, Huey RB, Sheldon KS, Ghalambor CK, Haak DC, et al. Impacts of climate warming on terrestrial ectotherms across latitude. Proc Natl Acad Sci. 2008 May 6;105(18):6668–72. doi: 10.1073/pnas.0709472105 18458348 PMC2373333

[94] Yom-Tov Y, Geffen E. Recent spatial and temporal changes in body size of terrestrial vertebrates: probable causes and pitfalls. Biol Rev. 2011;86(2):531–41. doi: 10.1111/j.1469-185X.2010.00168.x 21070587

