# Supplementary material for: Challenging ecogeographical rules: Phenotypic variation in the Mountain Treeshrew (Tupaia montana) along tropical elevational gradients
Source: PLoS One. 2022 Jun 17;17(6):e0268213. doi: 10.1371/journal.pone.0268213 (PMC9205479; doi:10.1371/journal.pone.0268213)
Supplement: S1 File — Methods, results and figures of dataset D2 statistical analyses, figure of body condition, and pictures highlighting differences in fur of Mountain Treeshrews (Tupaia montana) along elevation. (DOCX) [file pone.0268213.s002.docx]

**Challenging ecogeographical rules: phenotypic variation in the Mountain Treeshrew (*Tupaia montana*) along tropical elevational gradients**

Arlo Hinckley, Ines Sanchez-Donoso, Mar Comas, Miguel Camacho-Sanchez, Melissa T. R. Hawkins, Noor Haliza Hasan and Jennifer A. Leonard

# S1 File. Appendix

# S1 File. Appendix Fig 1. Illustration of the four variables measured on the skulls of *Tupaia montana*.


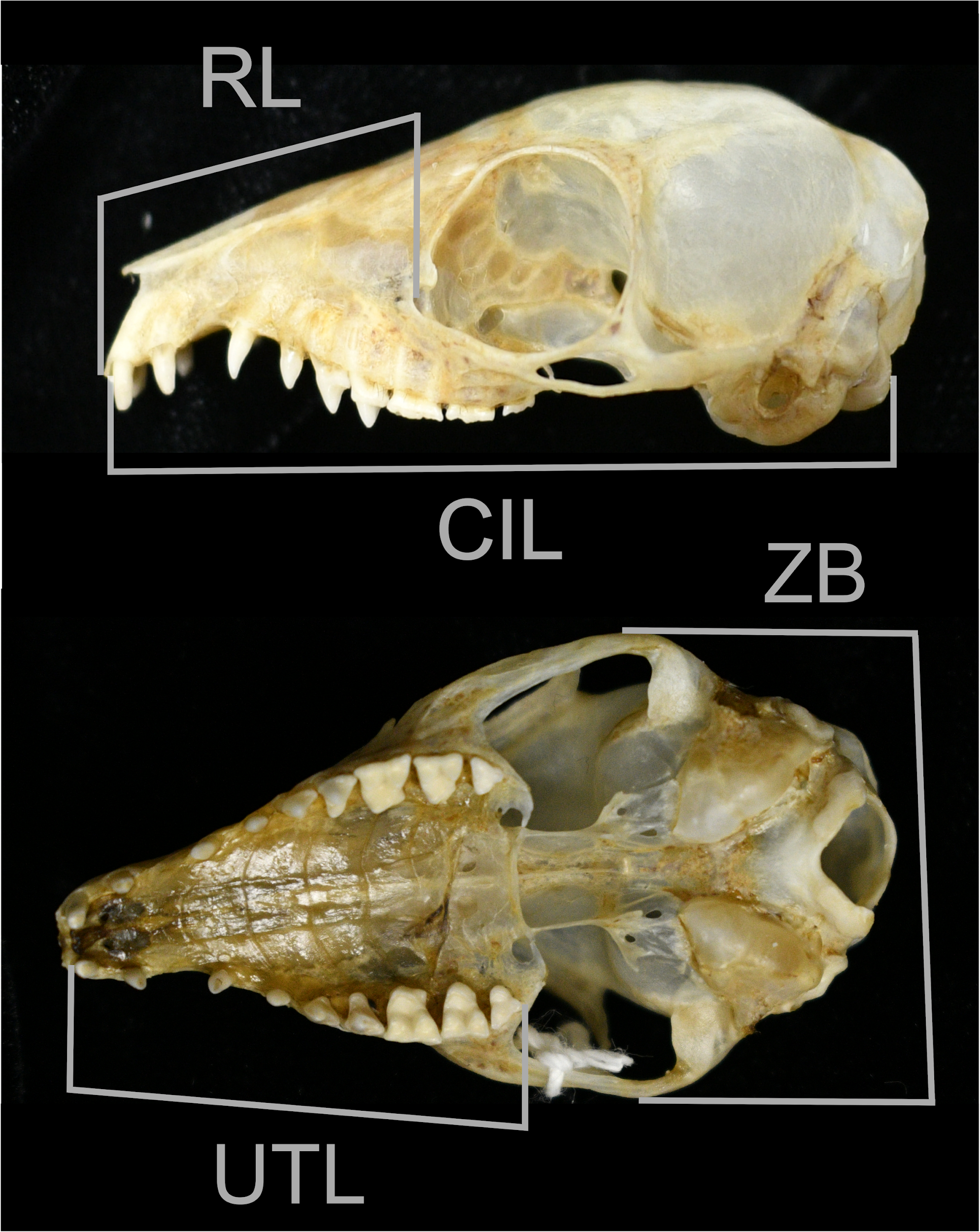


# Bergmann’s rule for D4

Most results regarding body size from the larger D4 dataset (adults aged by internal or external characters plus all individuals from Sabah Museum collection, for which we did not have information regarding age, N=374, pregnant females were excluded) support the results obtained with the less error-prone but smaller datasets analysed in the main text (**S1 File. Appendix Fig 2**).

Weight followed a significant U-shaped distribution along elevation (*F*_1,250_=8.0402 , *P*=0.0050), with non-significant differences between sexes (*F*_1,250_=1.5225, *P*=0.2184), as observed with the smaller D1 (weight model *R^2^_adj_*=0.1847, *F*_8,250_=8.305). A significant tendency to increase in weight was observed over the years (*F*_1,250_=16.9188, *P*=0.0001). Significant differences were observed among mountains, probably due to the non-balanced sample size among mountains (*F*_4,250_=7.6966, *P*< 0.00001).

Head-body length followed a significant U-shaped distribution along elevation (*F*_1,247_=21.8334, *P*< 0.00001), that was not detected in the main text analyses with the smaller sample size of D2 (head-body model *R^2^_adj_*=0.1613, *F*_8,247_=7.132), with no significant differences between sexes (*F*_1,247_=1.2039, *P*=0.2736). The same significant trend to increase along the years was confirmed, as shown in weight (*F*_1,247_=9.3297, *P*=0.0025), and significant differences were identified among mountains (*F*_4,247_=6.7557, *P*<0.0001)

**S1 File. Appendix Fig 2**. Distribution of (A and B) weight (in g) and (C and D) head-body (in mm) of D4 individuals over the elevational gradient studied. Vegetation zonation has been specified with vertical dotted lines. In A and C: black dots represent females, gray dots represent males. Lines show the relationship of the dependent variable with elevation. In B and D: coloration corresponds to the year of collection.


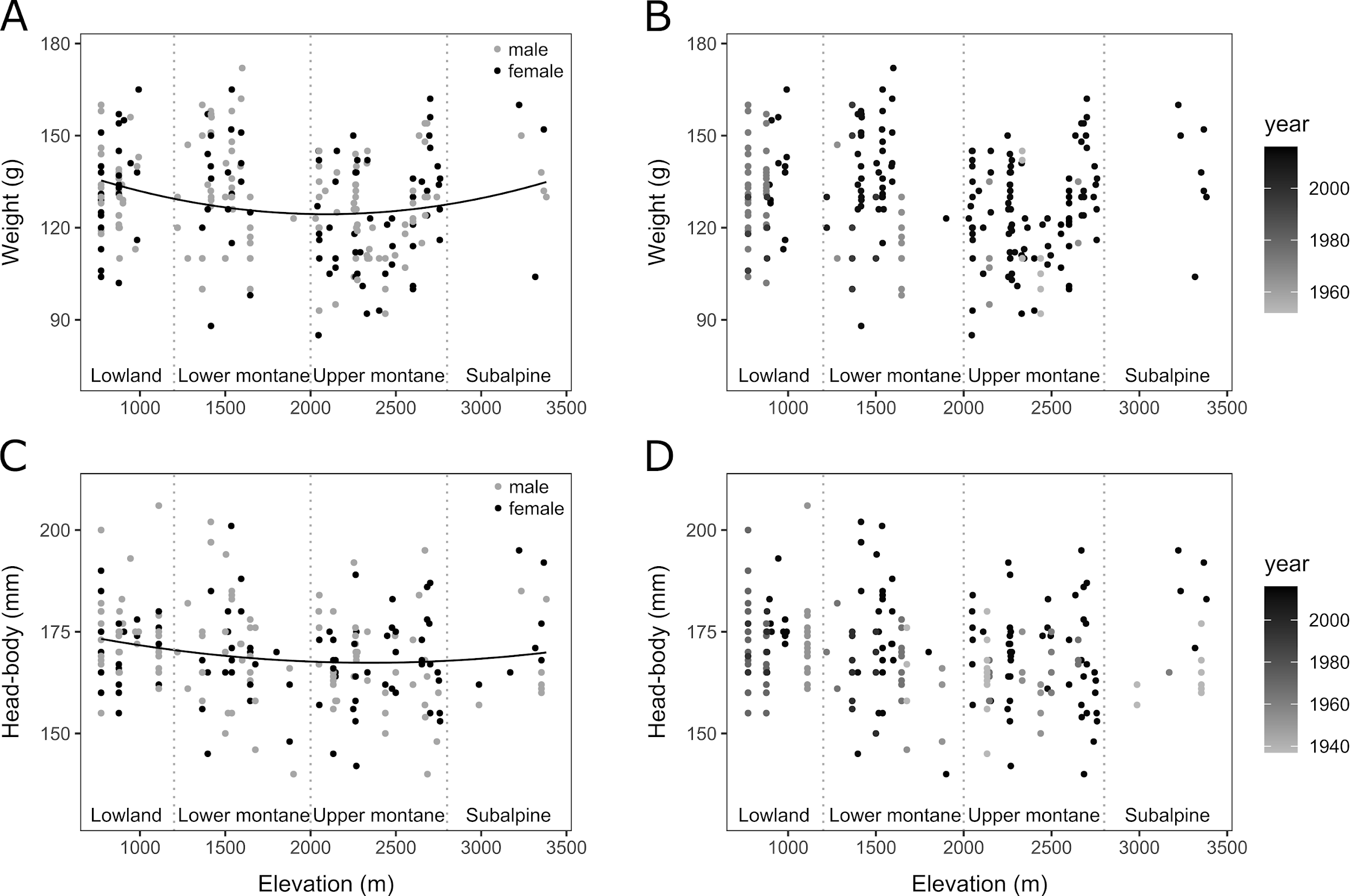


# Allens’s rule for D4 and D5

# External measurements

The results of the analyses of tail (TL) and ear length (EL) from D4 individuals supported the results obtained from the analyses of the smaller dataset shown in the main text (D2 and D3 individuals) while hindfoot length (HFL) from D5 individuals (N=167) did not mirror D3 analyses (**S1 File. Appendix Fig 3**).

Tail length (TL) significantly decreased with elevation following a linear trend (*F*_1,240_=33.4250, *P*< 0.00001), instead of the U-shaped pattern observed with the smaller dataset (D2) (TL model *R^2^_adj_*=0.2421, *F*_7,240_=12.27) (**S1 File. Appendix Fig 3A**). Differences in TL between sexes lost their significance (*F*_1,240_=0.9047, *P*=0.3425), in comparison to the significant sexual dimorphism observed in the smaller dataset (D2). We found significant differences in TL among mountains (*F*_4,240_=6.1624, *P*=0.0001) that were not significant in the smaller dataset analysis (D2), that corresponded to the significantly higher values observed in Maligan Range vs. Crocker Range (*posthoc Tukey test estimate*=11.037*, df*=240, *P*=0.0001), Mt. Kinabalu (*posthoc Tukey test estimate*=7.195*, df*=240, *P*=0.0177) and Mt. Tambuyukon (*posthoc Tukey test estimate*=10.877*, df*=240, *P*=0.0012) (Maligan Range mean TL=154 mm, SE=2.09, N=21; Kinabalu mean TL=147, SE=0.87, N=140; Tambuyukon mean TL=143 mm, SE=1.78, N=28; TL values averaged over sexes). Head-body length (HBL) had a significant effect over TL (*F*_1,240_=4.0880, *P*=0.0443) (TL model *R^2^_adj_*=0.2421, *F*_7,240_=12.27).

Hindfoot length (HFL) did not follow a significant trend over elevation (*F*_1,142_=0.8719, *P*=0.3520), contrary to the results obtained when analysing HFL with the less error-prone dataset (D3). The trend observed in HFL with this larger dataset (D5, **S1 File. Appendix Fig 3B, C**) might be biased due to a highly significant temporal effect (*F*_1,142_=41.9069, *P* < 0.00001). Males had significantly longer HFL than females (*F*_1,142_=4.5493, *P*=0.0347; female mean HFL =40.9 mm, *SE*=0.52, *N*=66; male mean HFL = 41.7 mm, *SE*=0.50, *N*=83; HFL means averaged over mountains), mirroring the smaller dataset. However, as stated in the main text, statistical significance does not imply biological significance. The difference among sexes in HFL was less than 1 mm, which is this measurement error. Significant differences were still found among mountains, probably due to the non-balanced dataset (*F*_2,142_=3.4083, *P*=0.0358, **S1 File. Appendix Fig 3B, C**), and the effect of head-body length (HBL) over HFL was also significant (*F*_1,142_=10.3118, *P* =0.0016) (HFL model *R^2^_adj_*=0.2998, *F*_6,142_=11.56).

Ear length (EL) did not show a significant trend over elevation (*F*_2,243_=1.8260, *P*= 0.1779) and sexual dimorphism was not significant (*F*_1,243_= 3.3808, *P*= 0.06718), as observed in the smaller dataset (D3, **S1 File. Appendix Fig 3D**) A significant trend to decrease along the years was detected (*F*_1,243_=36.5292, *P*<0.00001) (**S1 File. Appendix Fig 3E**). The effect of HBL over EL was not significant (*F*_1,243_= 1.5569, *P*= 0.2133) (EL model *R^2^_adj_*= 0.1491, *F*_4,243_= 11.82).

**S1 File. Appendix Fig 3.** Distribution of (A) tail, (B and C) hindfoot and (D and E) ear length (all three were measured in mm) of D4 and D5 individuals (the last dataset only for hindfoot analyses) over the elevational and temporal gradient studied. Vegetation zonation has been specified with vertical dotted lines. Black dots: females; grey dots: males. Lines show the relationship of the dependent variable with elevation, for both sexes.


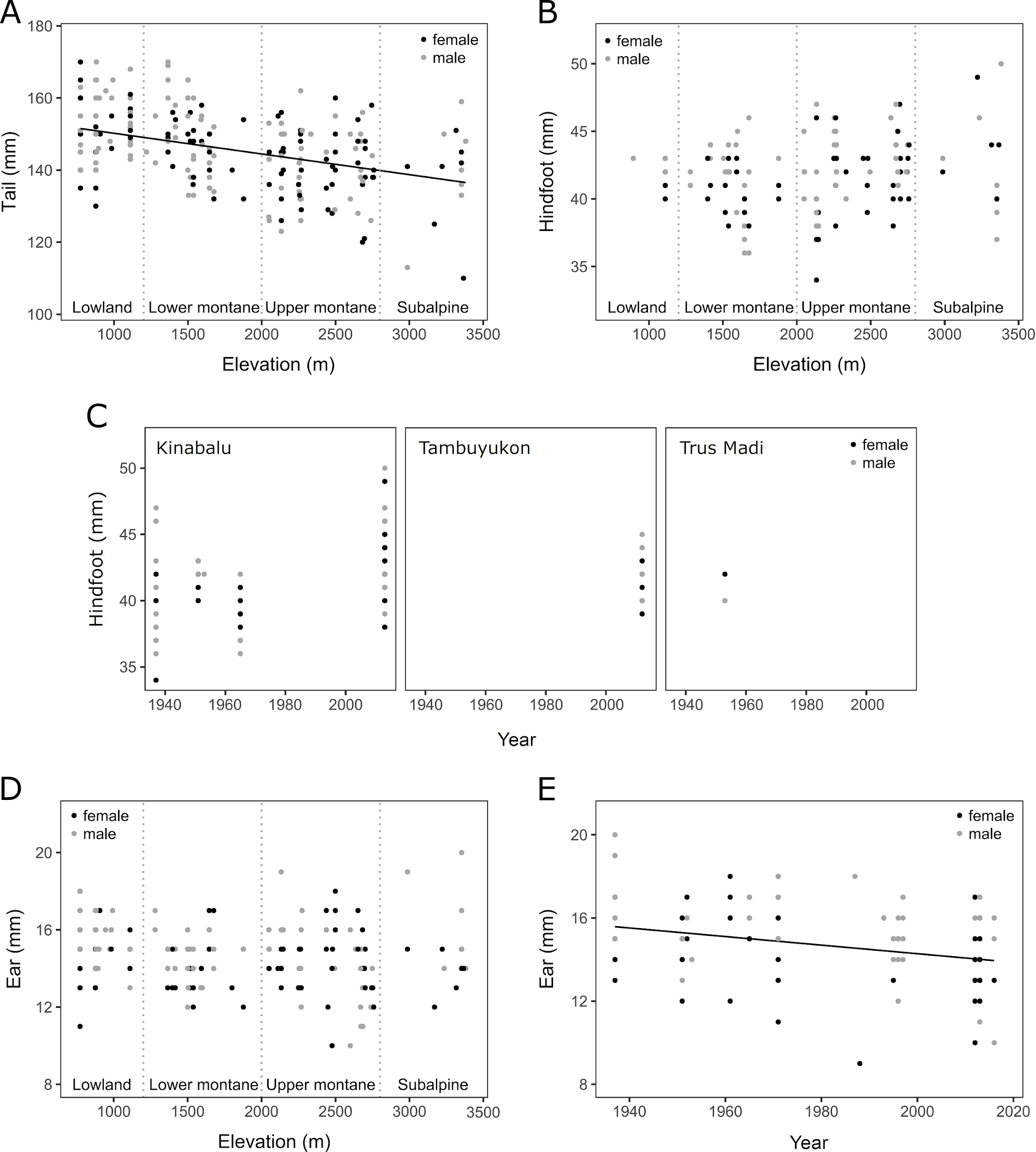


## Body Condition

**S1 File. Appendix Fig 4**. Distribution of body condition (Scaled Mass Index, SMI) of D2 individuals over the elevational gradient studied. Vegetation zonation has been specified with vertical dotted lines. Black dots: females; gray dots: males.


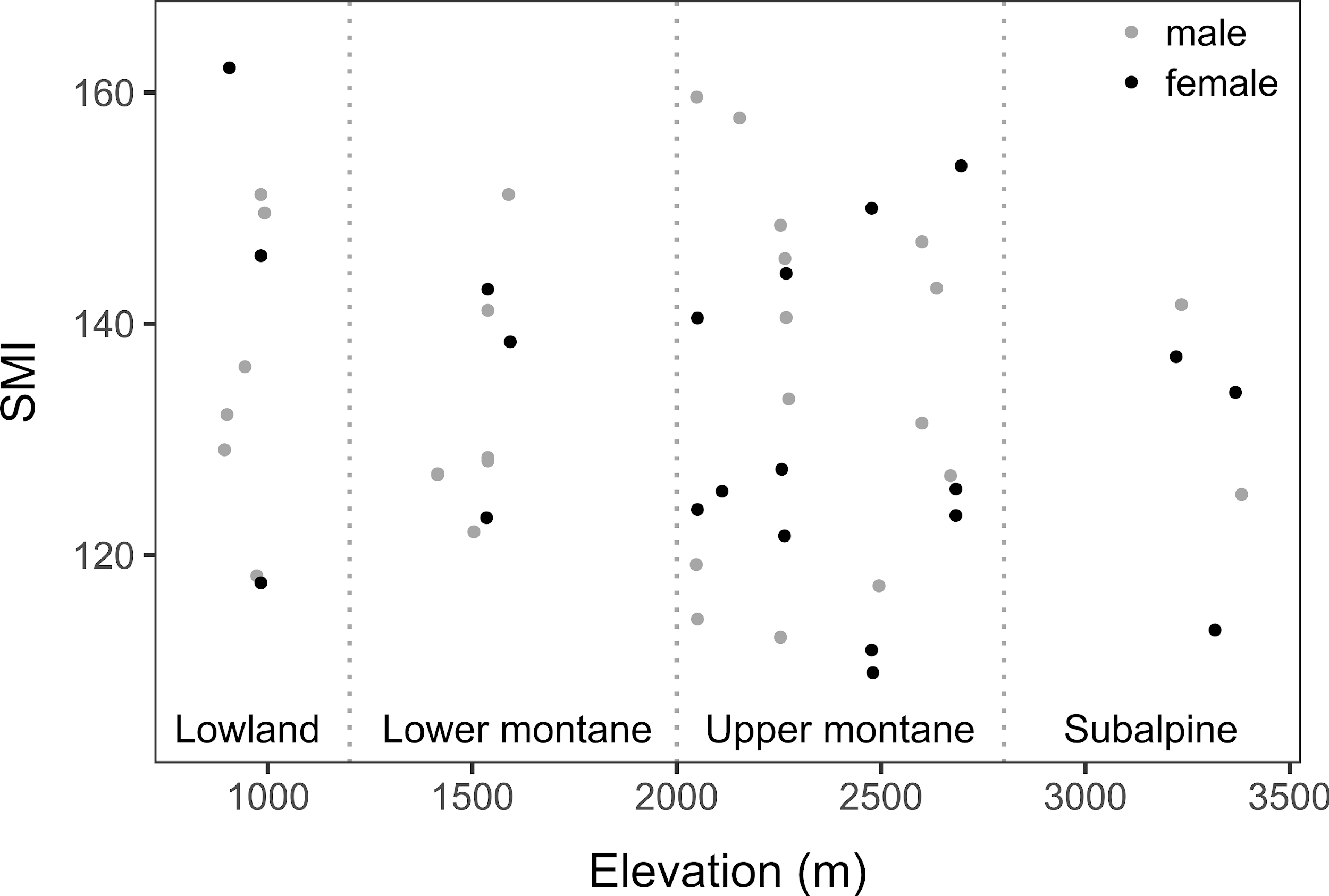


**S1 File. Appendix Fig 5.** Dorsum pelage of Mountain Treeshrew (*Tupaia montana)* at scapula. Both pictures were taken at the same scale. The upper one belongs to specimen EBD31346M from Tambuyukon at 836 m while the bottom one is EBD31360M from Mt. Tambuyukon at 2051 m. A conspicuous increase in hair density can be observed in the higher elevation individual’s fur.


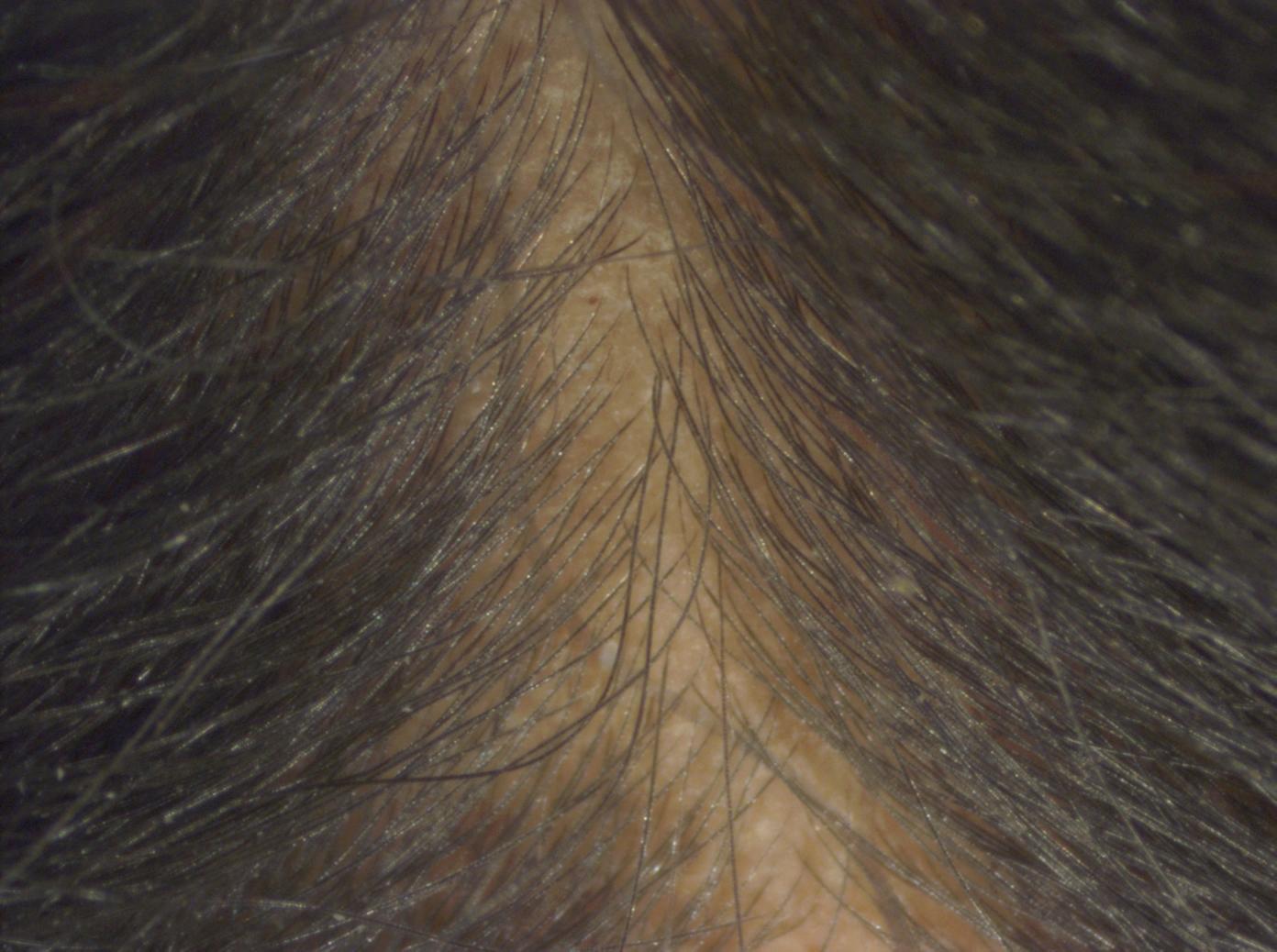


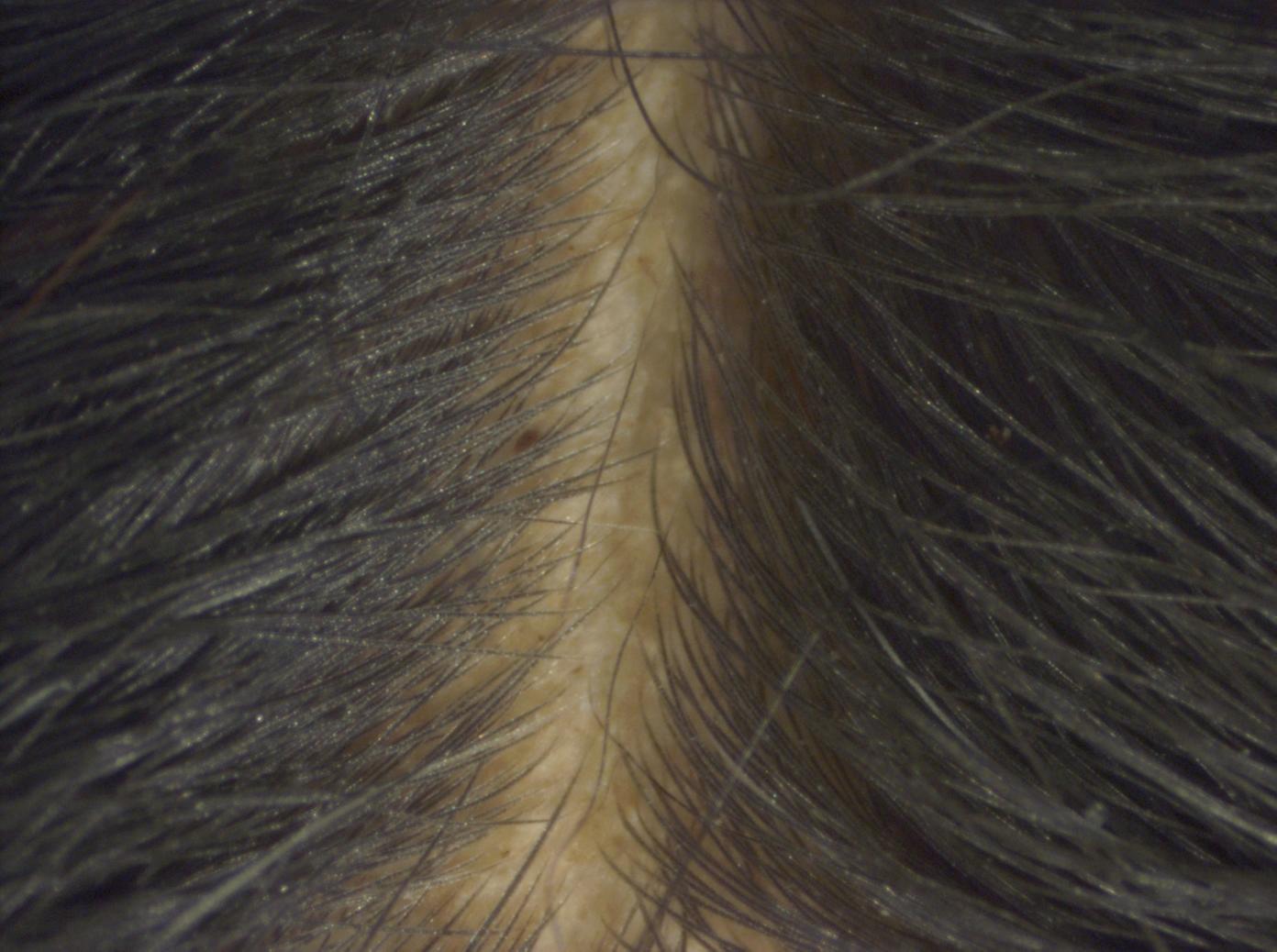


# Statistical models

Formulation of the linear models adjusted to the different datasets to analyze the significance of the effects of elevation and other explanatory variables over the several response variables studied are shown, as well as the coefficients obtained from the *F*-test used to evaluate significance of the effect of each explanatory variable.

We also include the sample size (*N*), the average and the standard error (*SE*) for W, HBL and CIL per vegetation zone (taking into account differences between sexes in CIL, since they were statistically significant). Vegetation zones were: lowland (< 1,200 m a.s.l.), lower montane (1,200-2,000 m a.s.l.), upper montane (2,000-2,800 m a.s.l.), subalpine (2,800-3,400 m a.s.l.).

**S1 File. Appendix Table 1.** **Statistical models from the main text - Bergmann’s rule**

**Weight (W) model, adjusted to D1:**

lm (formula = W ~ st_elevation + st_elevation^2^ + sex + year, data = D1)

**Head-body length (HBL) model, adjusted to D2:**

lm (formula = HBL ~ elevation + sex, data = D2)

**Condyle-incisive length** **(CIL) model, adjusted to D6:**

lm (formula = CIL ~ st_elevation + st_elevation^2^ + sex, data = D6)

|  |  | | Response variable | | |
| --- | --- | --- | --- | --- | --- |
|  |  |  | W | HBL | **CIL** |
| **Explanatory variables** | Elevation | *F* | - | 0.1862 | - |
|  |  | ***df*** | - | 1, 48 | - |
|  |  | *P* | - | 0.6680 | - |
|  | St. Elevation | *F* | 3.7270 | - | 24.9412 |
|  |  | ***df*** | 1, 79 | - | 1, 102 |
|  |  | *P* | 0.0571 | - | <0.00001 * |
|  | **St. Elevation^2^** | *F* | 4.6719 | - | 8.7565 |
|  |  | ***df*** | 1, 79 | - | 1, 102 |
|  |  | *P* | 0.0337 * | - | 0.0038 * |
|  | **Sex** | *F* | 0.0469 | 0.1496 | 6.9790 |
|  |  | ***df*** | 1, 79 | 1, 48 | 1, 102 |
|  |  | *P* | 0.8292 | 0.7006 | 0.0096 * |
|  | **Mountain** | *F* | - | - | - |
|  |  | ***df*** | - | - | - |
|  |  | *P* | - | - | - |
|  | **Year** | *F* | 17.3116 | - | - |
|  |  | ***df*** | 1, 79 | - | - |
|  |  | *P* | <0.0001 * | - | - |
| Whole model | | ***R^2^_adj_*** | 0.2498 | -0.0326 | 0.2363 |
|  |  | ***F*** | 7.909 | 0.2104 | 11.83 |
|  |  | *df* | 4, 79 | 2, 48 | 3, 102 |
| St. Elevation: standardized elevation; St. Elevation^2^: squared standardized elevation; *F*: F-statistic; *df*: degrees of freedom; *P*: p-value; *R^2^_adj_*: adjusted R^2^ of the model; *: significant p-value; -: no coefficient since that explanatory variable was not included in the model. | | | | | |

| **Variable** | **Vegetation zone** | ***N*** | **Average** | ***SE*** |
| --- | --- | --- | --- | --- |
| W | Lowland | 9 | 136.0 | 5.0 |
|  | Lower_montane | 24 | 129.1 | 3.5 |
|  | Upper_montane | 45 | 122.8 | 2.1 |
|  | Subalpine | 6 | 139.0 | 8.2 |
| HBL | Lowland | 9 | 178.0 | 2.1 |
|  | Lower_montane | 11 | 188.5 | 3.2 |
|  | Upper_montane | 26 | 174.8 | 2.0 |
|  | Subalpine | 5 | 185.2 | 4.2 |
| **CIL** (females) | Lowland | 17 | 45.2 | 0.3 |
|  | Lower_montane | 5 | 43.4 | 0.6 |
|  | Upper_montane | 20 | 43.8 | 0.2 |
|  | Subalpine | 3 | 44.3 | 0.6 |
| **CIL** (males) | Lowland | 26 | 45.3 | 0.2 |
|  | Lower_montane | 12 | 45.4 | 0.2 |
|  | Upper_montane | 20 | 44.4 | 0.3 |
|  | Subalpine | 3 | 44.8 | 0.3 |
| *N*: sample size; *SE*: standard error | | | | |

**S1 File. Appendix Table 2.** **Statistical models from the main text – Allen’s rule**

**Tail length (TL) model, adjusted to D2:**

lm (formula = TL ~ st_elevation + st_elevation^2^ + sex + HBL, data = D2)

**Hindfoot length (HFL)model, adjusted to D3:**

lm (formula = HFL ~ elevation + sex + mountain + HBL, data = D3)

**Ear length (EL) model, adjusted to D3:**

lm (formula = EL ~ elevation + sex + HBL, data = D3)

|  |  | | Response variable | | |
| --- | --- | --- | --- | --- | --- |
|  |  |  | **TL** | **HFL** | EL |
| **Explanatory variables** | Elevation | *F* | - | 12.954 | 0.0806 |
|  |  | ***df*** | - | 1, 30 | 1, 30 |
|  |  | *P* | - | 0.0011 * | 0.7784 |
|  | **St. Elevation** | *F* | 21.7643 | - | - |
|  |  | ***df*** | 1, 46 | - | - |
|  |  | *P* | <0.0001 * | - | - |
|  | **St. Elevation^2^** | *F* | 6.2188 | - | - |
|  |  | ***df*** | 1, 46 | - | - |
|  |  | *P* | 0.0163 * | - | - |
|  | **Sex** | *F* | 4.8827 | 7.639 | 0.0670 |
|  |  | ***df*** | 1, 46 | 1, 30 | 1, 30 |
|  |  | *P* | 0.0321 * | 0.0097 * | 0.7975 |
|  | **Mountain** | *F* | - | 11.261 | - |
|  |  | ***df*** | - | 1, 30 | - |
|  |  | *P* | - | 0.0022 * | - |
|  | HBL | *F* | 3.1997 | 11.669 | 0.1985 |
|  |  | ***df*** | 1, 46 | 1, 30 | 1, 30 |
|  |  | *P* | 0.0802 | 0.0018 * | 0.6591 |
| Whole model | | *R^2^_adj_* | 0.353 | 0.5665 | -0.08947 |
|  |  | ***F*** | 7.821 | 12.11 | 0.09663 |
|  |  | *df* | 4, 46 | 4, 30 | 3, 30 |
| St. Elevation: standardized elevation; St. Elevation^2^: squared standardized elevation; *F*: F-statistic; *df*: degrees of freedom; *P*: p-value; *R^2^_adj_*: adjusted R^2^ of the model; *: significant p-value; -: no coefficient since that explanatory variable was not included in the model. | | | | | |

**S1 File. Appendix Table 3. Statistical models from the main text – Diet-associated and insulation traits**

**Rostrum length (RL) model, adjusted to D6:**

lm (formula = RL ~ elevation + sex + CIL, data = D6)

**Zygomatic breadth (ZB) model, adjusted to D6:**

lm (formula = ZB ~ elevation + sex + year + CIL, data = D6)

**Upper tooth-row length (UTL) model, adjusted to D6:**

lm (formula = UTL ~ st_elevation + st_elevation^2^ + sex + mountain + CIL, data = D6)

|  |  | | Response variable | | |
| --- | --- | --- | --- | --- | --- |
|  |  |  | RL | ZB | **UTL** |
| **Explanatory variables** | Elevation | *F* | 6.1665 | 13.6604 | - |
|  |  | ***df*** | 1, 94 | 1, 101 | - |
|  |  | *P* | 0.0148 * | 0.0004 * | - |
|  | **St. Elevation** | *F* | - | - | 15.1360 |
|  |  | ***df*** | - | - | 1, 98 |
|  |  | *P* | - | - | 0.0002 * |
|  | **St. Elevation^2^** | *F* | - | - | 4.4334 |
|  |  | ***df*** | - | - | 1, 98 |
|  |  | *P* | - | - | 0.0378 * |
|  | **Sex** | *F* | 3.2581 | 7.3389 | 0.5648 |
|  |  | ***df*** | 1, 94 | 1, 101 | 1, 98 |
|  |  | *P* | 0.0743 | 0.0079 * | 0.45415 |
|  | **Mountain** | *F* | - | - | 4.2664 |
|  |  | ***df*** | - | - | 2, 98 |
|  |  | *P* | - | - | 0.0167 * |
|  | **Year** | *F* | - | 5.8175 | - |
|  |  | ***df*** | - | 1, 101 | - |
|  |  | *P* | - | 0.0177 * | - |
|  | **CIL** | *F* | 314.0722 | 33.5914 | 271.1278 |
|  |  | ***df*** | 1, 94 | 1, 101 | 1, 98 |
|  |  | *P* | < 0.00001 * | < 0.00001 * | < 0.00001 * |
| Whole model | | ***R^2^_adj_*** | 0.8127 | 0.3857 | 0.818 |
|  |  | ***F*** | 141.3 | 17.48 | 78.93 |
|  |  | *df* | 3, 94 | 4, 101 | 6, 98 |
| St. Elevation: standardized elevation; St. Elevation^2^: squared standardized elevation; *F*: F-statistic; *df*: degrees of freedom; *P*: p-value; *R^2^_adj_*: adjusted R^2^ of the model; *: significant p-value; -: no coefficient since that explanatory variable was not included in the model. | | | | | |

**S1 File. Appendix Table 4.** **Statistical models from Supplementary Data – Bergmann’s rule**

**Weight (W) model, adjusted to D4:**

lm (formula = W ~ st_elevation + st_elevation^2^ + sex + mountain + year, data = D4)

**Head-body length (HBL) model, adjusted to D4:**

lm (formula = HBL ~ st_elevation + st_elevation^2^ + sex + mountain + year, data = D4)

|  |  | | Response variable | |
| --- | --- | --- | --- | --- |
|  |  |  | W | HBL |
| **Explanatory variables** | St. Elevation | *F* | 15.8541 | 31.6310 |
|  |  | ***df*** | 1, 250 | 1, 247 |
|  |  | *P* | 0.0001 * | < 0.00001 * |
|  | St. Elevation^2^ | *F* | 8.0402 | 21.8334 |
|  |  | ***df*** | 1, 250 | 1, 247 |
|  |  | *P* | 0.0050 * | < 0.00001 * |
|  | **Sex** | *F* | 1.5225 | 1.2039 |
|  |  | ***df*** | 1, 250 | 1, 247 |
|  |  | *P* | 0.21839 | 0.2736 |
|  | **Mountain** | *F* | 7.6966 | 6.7557 |
|  |  | ***df*** | 4, 250 | 4, 247 |
|  |  | *P* | < 0.00001 * | < 0.0001* |
|  | **Year** | *F* | 16.9188 | 9.3297 |
|  |  | ***df*** | 1, 250 | 1, 247 |
|  |  | *P* | 0.0001 * | 0.0025 * |
| Whole model | | *R^2^_adj_* | 0.1847 | 0.1613 |
|  |  | ***F*** | 8.305 | 7.132 |
|  |  | *df* | 8, 250 | 8, 247 |
| St. Elevation: standardized elevation; St. Elevation^2^: squared standardized elevation; *F*: F-statistic; *df*: degrees of freedom; *P*: p-value; *R^2^_adj_*: adjusted R^2^ of the model; *: significant p-value. | | | | |

| **Variable** | **Vegetation zone** | ***N*** | **Average** | ***SE*** |
| --- | --- | --- | --- | --- |
| W | Lowland | 71 | 133.2 | 1.6 |
|  | Lower_montane | 70 | 130.4 | 2.2 |
|  | Upper_montane | 111 | 123.4 | 1.6 |
|  | Subalpine | 7 | 138.0 | 7.0 |
| HBL | Lowland | 92 | 172.1 | 0.9 |
|  | Lower_montane | 61 | 170.1 | 1.7 |
|  | Upper_montane | 89 | 166.7 | 1.1 |
|  | Subalpine | 14 | 171.6 | 3.4 |
| *N*: sample size; *SE*: standard error | | | | |

**S1 File. Appendix Table 5.** **Statistical models from Supplementary Data – Allen’s rule**

**Tail length (TL) model, adjusted to D4:**

lm (formula = TL ~ elevation + sex + mountain + HBL, data = D4)

**Hindfoot length (HFL) model, adjusted to D5:**

lm (formula = HFL ~ elevation + sex + mountain + year + HBL, data = D5)

**Ear length (EL) model, adjusted to D4:**

lm (formula = EL ~ elevation + sex + year + HBL, data = D4)

|  |  | | Response variable | | |
| --- | --- | --- | --- | --- | --- |
|  |  |  | TL | HFL | EL |
| **Explanatory**  **variables** | Elevation | *F* | 33.4250 | 0.8719 | 1.8260 |
|  |  | ***df*** | 1, 240 | 1, 142 | 1, 243 |
|  |  | *P* | < 0.00001 * | 0.3520 | 0.1779 |
|  | **Sex** | *F* | 0.9047 | 4.5493 | 3.3808 |
|  |  | ***df*** | 1, 240 | 1, 142 | 1, 243 |
|  |  | *P* | 0.3425 | 0.0347 * | 0.0672 |
|  | **Mountain** | *F* | 6.1624 | 3.4083 | - |
|  |  | ***df*** | 4, 240 | 2, 142 | - |
|  |  | *P* | 0.0001 * | 0.0358 * | - |
|  | **Year** | *F* | - | 41.9069 | 36.5292 |
|  |  | ***df*** | - | 1, 142 | 1, 243 |
|  |  | *P* | - | < 0.00001 * | < 0.00001 * |
|  | **HBL** | *F* | 4.0880 | 10.3118 | 1.5569 |
|  |  | ***df*** | 1, 240 | 1, 142 | 1, 243 |
|  |  | *P* | 0.0443 * | 0.0016 * | 0.2133 |
| **Whole model** | | *R^2^_adj_* | 0.2421 | 0.2998 | 0.1491 |
|  |  | ***F*** | 12.27 | 11.56 | 11.82 |
|  |  | *df* | 7, 240 | 6, 142 | 4, 243 |
| *F*: F-statistic; *df*: degrees of freedom; *P*: p-value; *R^2^_adj_*: adjusted R^2^ of the model; *: significant p-value; -: no coefficient since that explanatory variable was not included in the model. | | | | | |

**S1 File. Appendix Table 6.** **Statistical model of body condition (SMI)**

**SMI model, adjusted to D2:**

lm (formula = SMI ~ elevation + sex + mountain + year, data=D2)

|  | | | Response variable |
| --- | --- | --- | --- |
|  |  |  | SMI |
| **Explanatory**  **variables** | Elevation | *F* | 1.2575 |
|  |  | ***df*** | 1, 45 |
|  |  | *P* | 0.2681 |
|  | **Sex** | *F* | 0.0856 |
|  |  | ***df*** | 1, 45 |
|  |  | *P* | 0.7712 |
|  | **Mountain** | *F* | 0.8265 |
|  |  | ***df*** | 2, 45 |
|  |  | *P* | 0.4441 |
|  | **Year** | *F* | 1.1595 |
|  |  | ***df*** | 1, 45 |
|  |  | *P* | 0.2873 |
| Whole model | | *R^2^_adj_* | -0.01903 |
|  |  | ***F*** | 0.8132 |
|  |  | *df* | 5, 45 |
| *F*: F-statistic; *df*: degrees of freedom; *P*: p-value; *R^2^_adj_*: adjusted R^2^ of the model. | | | |
